# SVXplorer: Three-tier approach to identification of structural variants via sequential recombination of discordant cluster signatures

**DOI:** 10.1371/journal.pcbi.1007737

**Published:** 2020-03-17

**Authors:** Kunal Kathuria, Aakrosh Ratan

**Affiliations:** 1 Center for Public Health Genomics, University of Virginia, Charlottesville, Virginia, United States of America; 2 Department of Public Health Sciences, University of Virginia, Charlottesville, Virginia, United States of America; Carnegie Mellon University, UNITED STATES

## Abstract

The identification of structural variants using short-read data remains challenging. Most approaches that use discordant paired-end sequences ignore non-trivial signatures presented by variants containing 3 breakpoints, such as those generated by various copy-paste and cut-paste mechanisms. This can result in lower precision and sensitivity in the identification of the more common structural variants such as deletions and duplications. We present SVXplorer, which uses a graph-based clustering approach streamlined by the integration of non-trivial signatures from discordant paired-end alignments, split-reads and read depth information to improve upon existing methods. We show that SVXplorer is more sensitive and precise compared to several existing approaches on multiple real and simulated datasets. SVXplorer is available for download at https://github.com/kunalkathuria/SVXplorer.

## Author summary

Structural variants (SVs) that include duplications, deletions and inversions of large blocks of DNA sequence account for the greatest share of total nucleotide differences between individuals. Most methods to identify SVs focus on deletions, duplications, and inversions which can be identified by the integration of information from coverage and insert length of aligned reads around the breakpoints. These methods either ignore signatures from other non-trivial rearrangements or represent them as a set of novel adjacencies (breakends) in the output to be processed separately. Here, we show that precise accounting of such signatures and using improved methods for clustering and variant filtering markedly enhance the precision and sensitivity of SV calls such as deletions and duplications, and allow for detection of several other categories of SVs.

This is a *PLOS Computational Biology* Methods paper.

## Introduction

Structural variants (SVs), which include regions of genomic imbalances called copy number variants (CNVs) and balanced rearrangements such as inversions, account for the majority of varying bases in the human genome [1]. SVs are more common in regions with segmental duplications and have been associated with phenotypes ranging from sensory perception [2] to genomic disorders such as the velocardiofacial [3] and Smith-Margenis syndromes [4]. The discovery and genotyping of these variants remain challenging due to their proximity to repeats, limitations of the alignment algorithms, large non-Gaussian spread in insert size, and the short read lengths typically used in sequencing.

**Fig 1:**
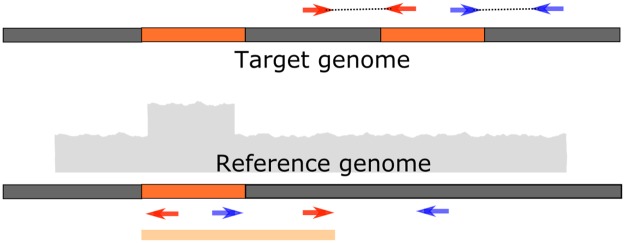
Duplication breakpoints. The breakpoints of the duplicated segment might be incorrectly identified as shown by the light orange segment at the bottom, if the overlap of signatures from an insertion via ‘copy-paste’ are ignored. If read-depth filters do catch the false calls, the caller would miss the variant entirely as shown in the simulations.

More than 40 short-read SV callers have been published since 2010 [5]. SV identification from short-reads relies on detecting changes in read-depth (RD), clusters of discordantly aligned paired-end (PE) reads or split reads (SR), or *de novo* genome assembly. Earlier methods were developed to harness evidence from one of these sources, but more recent methods such as LUMPY [6], TIDDIT [7], and TARDIS [8] integrate multiple sources and typically outperform earlier methods [5]. Despite these developments, SV callers have varying accuracy for different classes of SVs, and most of them employ specifically designed heuristics for the identification of specific SV types [9–11]. Most of them focus on the detection of signatures of individual SV types, often ignoring 3-breakpoint SVs and their signatures. However, ignoring those signatures often leads to incorrect identification or annotation of common SVs such as deletions, duplications, and inversions. For example, in Fig 1, ignoring the overlap of signatures from the copy-paste insertion leads to identification of incorrect breakpoints or the wrong SV type in the region. Even if read-depth is used to discover the duplicated segment, such calls will be filtered away by the caller if it considers discordant read-pairs, but does not account for overlaps of the discordant pairs.

Transposable elements represent the majority of large insertions in the human genome, and specialized methods have been developed to detect them [12, 13]. These methods are required to handle the repetitive nature of these elements, which arise due to repeated copy-and-paste operations through target-primed reverse transcription (TPRT) [14]. But even when these transposable elements are not identified accurately, probabilistic models that do not account for them end up with a large number of false-positive as well as false-negative SV calls [15]. Analysis of the inversion calls from the 1000 genomes project [16] highlights the importance of joint identification of all calls in SV detection. As discussed in Soylev et al. [15], 54% of the predicted inversions reported in the 1000 Genomes project are inverted duplications that are incorrectly identified by callers which do not consider overlapping evidence from discordant pairs and do not call the SV events simultaneously.

We have developed SVXplorer, which uses an efficient, comprehensive 3-tier approach of sequentially using discordant paired-end (PE) alignment, split-read (SR) alignment and read-depth (RD) information to identify multiple SV types while progressively weeding out unlikely candidates. Using a combination of probabilistic and combinatorial approaches, SVXplorer shows marked improvement in comparison to several other popular SV callers on both simulated and real human datasets. It uses Bayesian inference to calculate the probability that two aligned fragments support the same variant based on a precise binning of sampled insert length values. This posterior probability is scaled and used as an edge-weight in a maximal-clique-enumeration framework that is similar to CLEVER [17]. Another unique feature of SVXplorer is its exhaustive analysis of cluster signatures to group clusters into two-breakpoint variants (e.g. inversions) and three-breakpoint variants (e.g. copy- and cut- paste insertions) for both PE and SR. SVXplorer implements this progressive variant formation by first combining existing PE clusters appropriately into variants, updating these variants by incorporating SR signatures as seen in each PE variant region, and grouping the remaining clusters of SR alignments into new 2- and 3- breakpoint variants. The signatures considered by SVXplorer include the ones for copy-paste and cut-paste insertion mechanisms (including inverted transcriptions), inversions, tandem duplications and other variants for varying cluster sizes as explained in the following sections.

SVXplorer dynamically calculates PE and SR alignment-support thresholds based on the sequencing read-depth rather than requiring a fixed number of supporting reads for all data-sets. It also corroborates SVs using local read-depth information from mappable and regionally reliable bases. SVXplorer’s general approach to SV-calling is to ensure a precise treatment at all levels, as shown by its handling of PE and SR inversions (see Results), as an example. The inclusion of these features in SVXplorer has demonstrated an improvement in the precision and sensitivity of calls for the common SV types over several other SV-callers. First, it exhibits superior performance for simulated data designed to compare various aspects of SV calling for all the tools, and particularly in the assessment of SVs arising from three-breakpoint insertion events. On data from two different libraries sequenced from the same cell line (NA12878) and the CHM1 haploid cell line, SVXplorer outperforms other methods in comparison to calls made using longer PacBio reads or using an ensemble caller. It also shows “highest” self-consistency for the two NA12878 libraries (though the consistency numbers across callers are not directly comparable as will be explained). With the same caveat, in sequences from a family trio, SVXplorer exhibits the “highest” fraction of calls that are shared between the child and the biological parents, while similarly identifying the “lowest” fraction of calls in the child that are not found in either of the parents. Among the methods that are compared, it also shows the highest agreement with an ensemble-based truth set for the child. Before moving onto following sections, the reader may find it helpful to read definitions/explanation of terminology used in the manuscript given at the beginning of the Methods section.

## Results

We compared SVXplorer (v0.0.4) to the following other structural variant callers: LUMPY (v0.2.11) [6], DELLY2 (v0.7.7) [18], MANTA (v1.6.0) [19], TIDDIT (v2.8.1) [7] and TARDIS (v1.0.6) [8]. These methods have been used in several large-scale studies including the 1000 Genomes Project, use more than one sources of evidence, and have been shown to be an improvement over most existing tools. We compared their performance to that of SVXplorer on both simulated and real human datasets.

LUMPY was run using the defaults in the ‘lumpy_express’ script with the exception of the ‘-x’ option which was used to supply a BED file of regions to be excluded from the analyses. These included regions with abnormally high coverage, the mitochondrial genome, the decoy genome and the genome of the Epstein-Barr virus (EBV) [6]. The detected SVs were then genotyped using SVTyper [20] and the calls were filtered to keep ones with at least one alternate allele. This genotyping improved the precision of calls made by LUMPY substantially. DELLY2 was run using the same parameters as used in Layer et al. (mapping quality threshold: 1, minimum support: 4) [6] and an additional BED file with known gaps in the human genome was provided to avoid spurious calls in those regions. MANTA was run using with the default mapping-quality (MQ) threshold and minimum support of 10 and 4 respectively, as in [19]. It was provided the same BED file as LUMPY to exclude regions that generate unreliable calls. TIDDIT was run using default specifications, and TARDIS was run with defaults in non-sensitive mode without mrFast realignment with the appropriate SONIC file. SVXplorer was run with its default parameter set using discordant paired-end (PE) alignments with mapping quality ≥ 1 and split-read (SR) alignments with mapping quality ≥ 10. SVXplorer was provided the same exclusion file as LUMPY, along with a BED file of mappable regions. For all tools, only the variants larger than 100 bps were kept for subsequent analyses and evaluation. These specifications were chosen for best overall performance on the human genome for each caller. None of the parameters were changed for any of the callers for any data set, except that no exclude file (or mappable regions file for SVXplorer) was used in processing of simulated datasets. Furthermore, TARDIS was run with ‘--no-interdup’ option for simulated datasets to avoid interspersed duplication clustering, as interspersed duplications were not simulated in this study.

There are significant differences in how the various methods report their calls in the output VCF file. LUMPY, DELLY, and MANTA do not identify 3-breakpoint events, and annotate the SVTYPE tag for each of the detected SVs with one of DUP (duplication), DEL(deletion), INV(inversion) and BND(breakend). TIDDIT annotates tandem-duplications as TDUP, and other duplication events are annotated as DUP. For duplication events such as those arising via copy-paste as shown in S3 Fig, TIDDIT records adjacencies between (a) x1 and x2, and (b) between x1 and x3, as BND events. For events arising via cut-paste insertions as shown in S4 Fig, it records an additional breakend adjacency between x2 and x3. TARDIS annotates duplications with a DUP:TANDEM for tandem duplications and a DUP:ISP for an interspersed duplication. TARDIS also annotates deletions as either DEL:ME for mobile-element deletions, and DEL for other deletion events. TARDIS does not output any breakend events but it uses the observed adjacencies to annotate the SVs as belonging to different variant classes. Our method, SVXplorer, records the adjacencies of S3 Fig as BND events between x1 and x2, and between x1 and x3 similar to TIDDIT. However, it records an additional DUP event in the VCF file between x2 and x3, and reports all 3 events successively in the output VCF unified by a common GROUPID tag. Cut-paste insertions are handled identically except that a DEL is recorded in the VCF file between x2 and x3 instead of a DUP. SVXplorer is the only tool that groups all detected events arising from 3-breakpoint insertions together, clearly identifying the source and the destination. TARDIS is the only other tool besides SVXplorer that annotates the source region of an insertion with the appropriate SV type.

### Simulated data

We first ran a haploid simulation wherein RSVSim [21] was used to simulate 2,000 deletions, 1,000 tandem-duplications, 200 inversions, 200 copy-paste insertions and 100 cut-paste insertions, each of sizes ranging uniformly at random from 100-10,000 bps in the human reference genome (GRCh37+decoy), placing breakpoints with a bias towards repeat regions and regions of high homology. We then simulated 100 bp Illumina paired-end sequences using wgsim (https://github.com/lh3/wgsim) with a specified mean insert length of 350 and standard deviation of 50, to an average coverage of 50X, and aligned them against the reference genome using BWA mem [22]. All the SV callers were then run on this dataset, and the results were converted to the BEDPE format. The variants were compared to the true breakpoints using BEDTools [23] ‘pairToPair’ allowing a tolerance of 200 bps.

With this simulation, we wanted to compare the methods in their ability to detect common SV types: deletions, duplications and inversions. For SVXplorer, we included calls that were tagged as DUP as duplications, and calls that were tagged as DEL as deletions. For TIDDIT, variants tagged as DUP or TDUP were considered as duplications, and variants tagged as DEL were considered in deletions. For TARDIS, variants tagged as DUP:TANDEM and DUP:ISP were included as duplications, whereas variants tagged as DEL or DEL:ME were included as deletions. For DELLY, MANTA, and LUMPY we considered all variants that were tagged as DUP or DEL in these assessments. We would also like to point out that both SVXplorer and TARDIS can detect and report on inverted duplications, but we did not include them in these comparisons as they are not currently supported by RSVSim, and the other approaches do not detect them.

**Fig 2:**
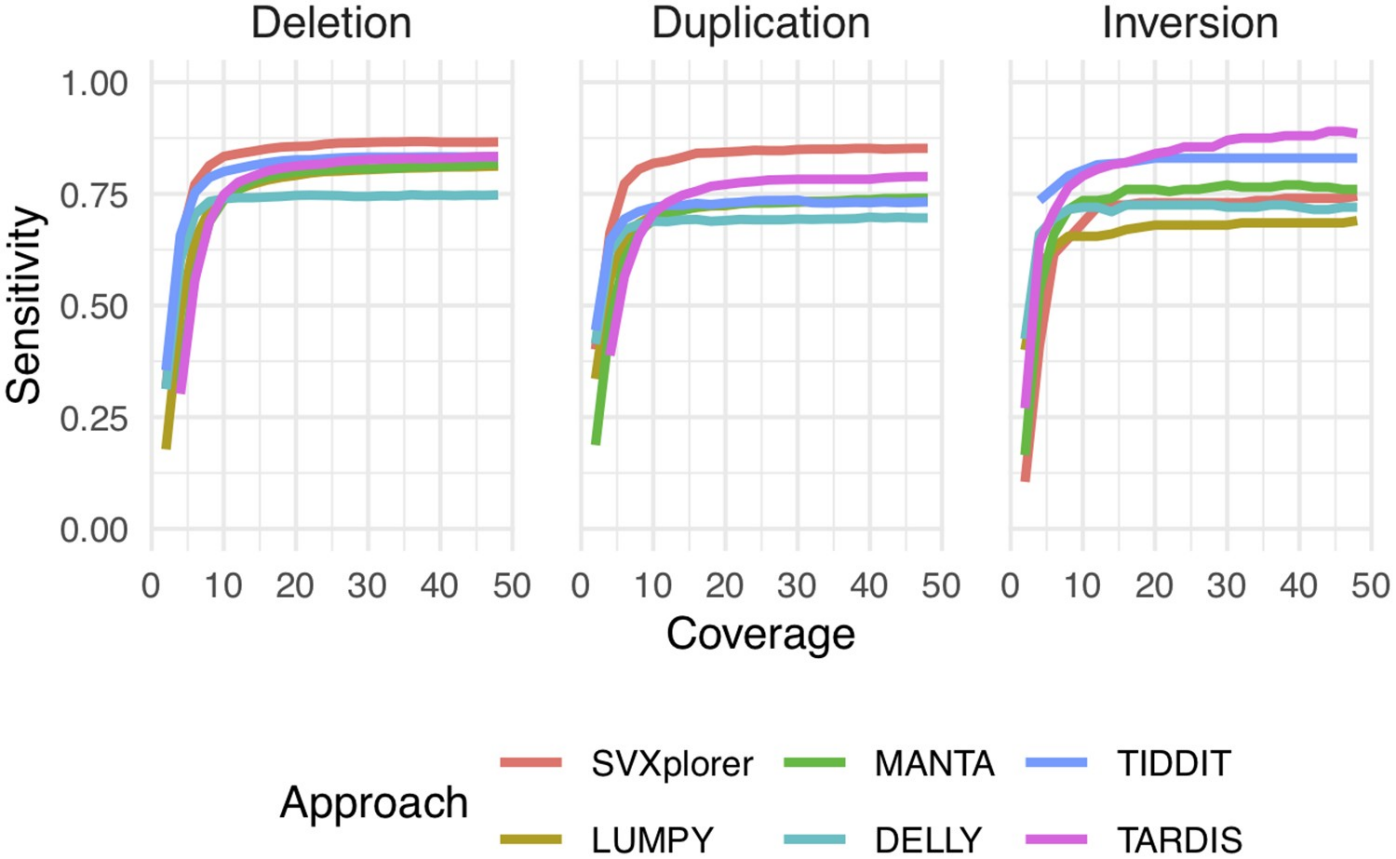
Simulation sensitivity. SVXplorer is more sensitive overall compared to the other approaches even at relatively low genome coverage, as assessed using this simulated dataset.

We then computed the sensitivity and precision in each variant category. The same simulation was repeated at coverages ranging from 2X to 48X in steps of 2X to assess how well the callers perform with varying sequenced information. The relative performance for all callers based on sensitivity of the calls is shown in Fig 2. SVXplorer has the highest sensitivity for deletions and duplications at all depths of coverage that were investigated. TARDIS and TIDDIT have the highest sensitivity for inversions closely followed by MANTA, SVXplorer and DELLY. The default specifications for SVXplorer are conservatively aimed at real data and they mandate that an inversion not be called unless evidence is seen at both ends of the variant in a stringent assessment (see Methods/S1 File for more details). In fact, as we show with real datasets, the number of inversion calls made by SVXplorer and LUMPY relative to other variants for real datasets are fewer, and much more in line with what is identified via long-read sequencing or ensembl calling. TARDIS was the only caller that called a high number of false-positive duplications and inversions (S10 Fig) for this simulated dataset. This false-positive rate for TARDIS can be significantly lowered by manual selection of parameters at each coverage. Two parameters, ‘--rp’ which is used to select the minimum number of supporting read pairs in initial clustering, and ‘--read-cluster’, which is used to select the number of clusters that a particular read can belong to, have a significant effect on the number of false-positive calls for TARDIS.

Even though TIDDIT, TARDIS, and SVXplorer can detect 3-breakpoint variants including translocations and interspersed duplications, they report these calls differently from each other. Most importantly, TIDDIT and TARDIS do not group the 2-breakpoint calls arising from 3-breakpoint insertions together like SVXplorer. TARDIS does not output all adjacencies, which makes it difficult to evaluate in comparison with SVXplorer and TIDDIT. It identifies the source location of the cut- or copy-paste insertion (and sometimes the corresponding insertion location as an “INS:ME” event). It also has a “POS2” field to identify the paste location for “DUP:ISP” calls, but it was always listed as being on the same chromosome (even for inter-chromosomal events) and not observed to be correct for the simulated variants. The reason for this is that TARDIS is designed to identify pre-annotated repeat elements in the human genome whereas the simulation places variants randomly throughout the genome. So we ran another simulation where we compared SVXplorer and TIDDIT for their ability to correctly detect cut- and copy-paste insertions. Again, we used RSVSim to generate an altered haploid target consisting of 1000 cut-paste and 1000 copy-paste insertions distributed throughout the genome with a bias towards repeat regions. The source region size of the insertion ranged uniformly from 100–100, 000 bps. The read simulation and alignment details as well as the stepwise simulated coverage were identical to Simulation 1. All tools were run in regular mode without any additional files or parameters. For this simulation, SVXplorer took 43 minutes and TIDDIT took 181 minutes to detect these variants at 50-fold coverage.

For evaluation, we considered that TIDDIT and SVXplorer recorded a true positive insertion when all 3 breakpoints were identified via BND adjacencies. BND events identifying some but not all of the 3 breakpoints were counted neither as true positive insertions nor false positives. Other SV types were also not counted as false positives if they correctly identified the 2 source region breakpoints (e.g. a deletion recorded for the source region of a cut-paste). All other calls counted as false positives. A slop of 300 was used for breakpoint evaluation due to the more complex nature of 3-breakpoint insertion calls. The plot of performance with respect to varying coverage is given in S11 Fig, which shows that at all coverages SVXplorer exhibits a higher sensitivity compared to TIDDIT for this dataset. SVXplorer also makes the advance over other tools of effectively unifying all adjacencies arising from a 3-breakpoint variant into a single SV call.

### Real data

We next applied SVXplorer along with the other callers to several real human sequencing datasets to evaluate its relative effectiveness under different conditions. Build 37 of the human genome (GRCh37+decoy) was used as the reference for all datasets. For predictive power, the callers were either evaluated against calls made using PacBio long reads, or those made using ensemble approaches such as Parliament [24]. Sensitivity, precision and F1 score were computed for all callers after removing calls less than 100 bps from both the call set and the truth set. A call in the “truth” set that overlaps a predicted call within a slop of 200 bps is defined as a true positive. Deletions were evaluated using events labelled as “DEL” in the VCF file for LUMPY, MANTA, DELLY, TIDDIT and SVXplorer. For TARDIS, we included the variants annotated as DEL or DEL:ME. Wherever possible, an assessment was made as to the self-consistency of calls made by each caller for related samples (different libraries or related individuals). The VCF files of variant calls made for these data can be downloaded from Zenodo (https://doi.org/10.5281/zenodo.3634028).

#### SVXplorer has high precision and sensitivity on CHM1 deletions

Performance was first evaluated on the CHM1 cell line, derived from a human haploid hydatidiform mole. We downloaded 40X coverage 2x100 bp Illumina WGS reads from the ENA short read archive (ENA accessions ERR1341794) and realigned it to the b37+decoy genome using BWA mem. We used the comprehensive set of structural variant calls released by Chaisson et al. [25] that are publicly available at http://eichlerlab.gs.washington.edu/publications/chm1-structural-variation as the truth set for this analysis. As we show in Table 1, SVXplorer outperforms the other methods in precision and F1 score in the identification of deletions. TARDIS has the highest sensitivity, followed by SVXplorer.

**Table 1:** Comparison of the various approaches based on CHM1 deletions.

	SVXplorer	LUMPY	DELLY	MANTA	TIDDIT	TARDIS
Calls	1759	1804	1116	1585	1386	3739
True-positives	1219	1174	397	958	852	**1265**
Sensitivity	27.3	26.3	8.9	21.5	19.1	**28.3**
Precision	**69.9**	65.0	35.5	60.4	61.5	33.8
F1-score	**39.3**	37.5	14.2	31.7	29.1	30.8

The “best” result for each metric is highlighted in bold.

**Fig 3:**
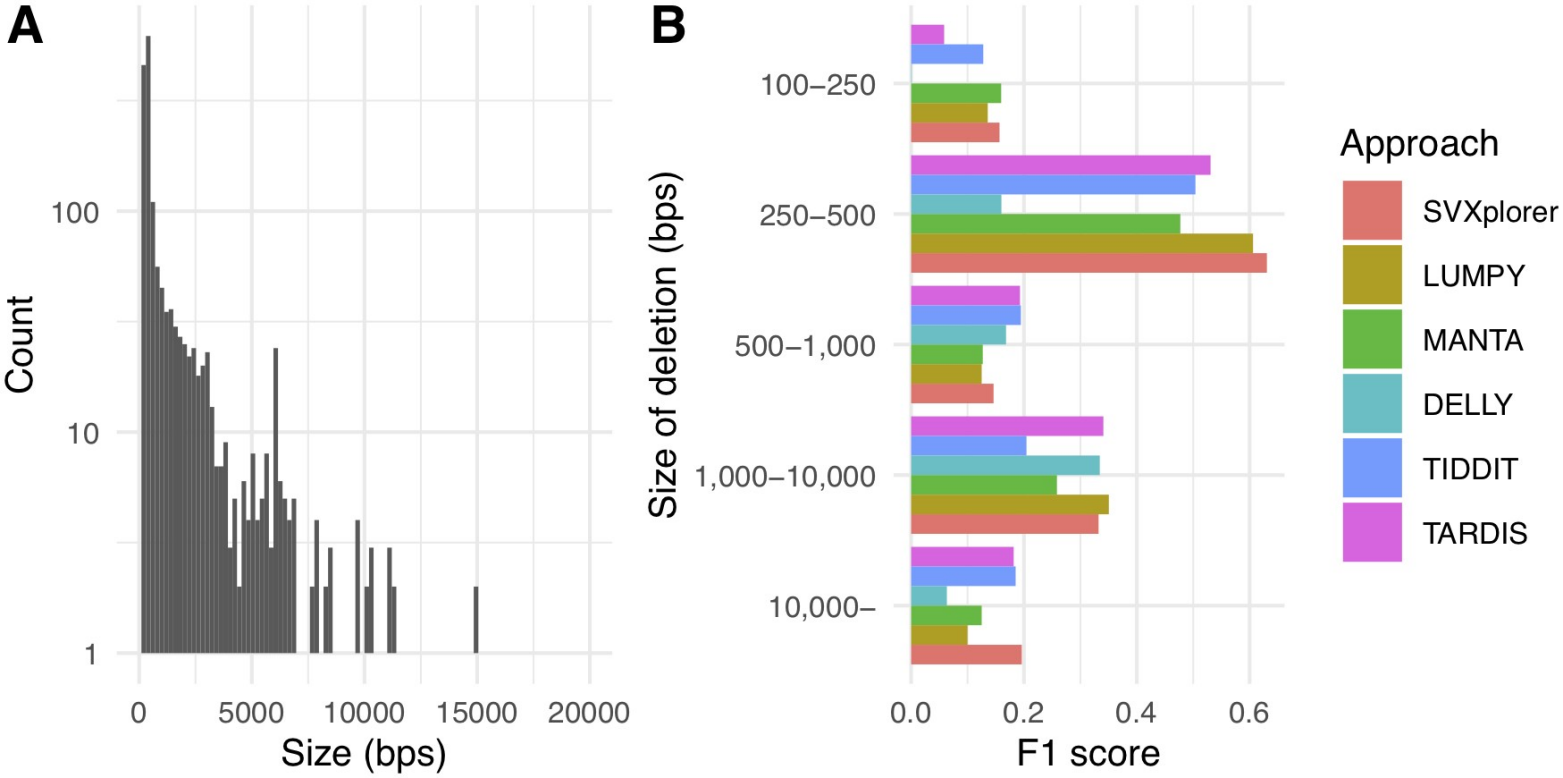
Size of detected deletions. (A) The size of deletions in CHM1 detected by SVXplorer. (B) Comparison of the SV callers based on F1 score for various deletion size intervals.

The truth set available for inversions had very few calls (33) to establish statistical significance in comparison across the different callers. Additionally, insertions are difficult to compare faithfully for sensitivity and precision because many callers classify them as generic breakend (BND) events. An enrichment analysis of the false-positive deletion made by SVXplorer using LOLA [26] against the UCSC genomic features database using a 1000 bps genomic segments as the background shows enrichment of regions covered by segmental duplications (OddsRatio: 1.47), CNVs in coriell cell line (OddsRatio: 1.4), LaminB1 Lads (OddsRatio: 1.27), nested repeats (OddsRatio: 1.06), and repeat masked regions (OddsRatio: 1.17). However, we would like to point out that 478 of the false-positives identified by SVXplorer are also called by LUMPY, 330 of them are also called by DELLY, and 431 of them are also called identified by MANTA and these calls be indicative of the limitations of short-read sequencing in determination of SV calls. Fig 3 shows the size distribution of deletions detected by SVXplorer, and we compare the F1 score of the various callers for the various sizes of deletions. Using the default parameters, DELLY does not identify small deletions less than 250 bps in this sample, and SVXplorer is consistently among the best callers for all strata in this dataset. We also show the size of the duplications and inversions detected by SVXplorer for this sample in S12 Fig.

#### SVXplorer achieves high self-consistency among multiple libraries

We next applied SVXplorer to two separate libraries for the well studied NA12878 cell line (accession numbers: ERR194147 and SRR505885) along with the other callers. The callers were evaluated for deletion calls against calls made using PacBio long reads that passed quality filters. We downloaded the calls made on the pacbio dataset from ftp://ftp-trace.ncbi.nlm.nih.gov/giab/ftp/data/NA12878/NA12878_PacBio_MtSinai. The callset was made using 7 different SV callers, and the calls made by at least 3 callers were considered to pass all quality filters. For both libraries SVXplorer exhibits better performance characteristics compared to other callers. The results are shown in Table 2.

**Table 2:** Comparison of deletion calls made by the various methods for NA12878. The “best” result for each metric is highlighted in bold.

Library		SVXplorer	LUMPY	DELLY	MANTA	TIDDIT	TARDIS
ERR194147	Calls	2724	2607	2407	2935	2264	2611
	True-positives	**2401**	2274	1867	2202	1874	2211
	Sensitivity	**64.9**	61.5	50.5	59.6	50.7	59.8
	Precision	**88.6**	87.2	77.5	75.1	82.8	84.7
	F1-score	**74.9**	72.1	61.2	66.4	62.9	70.1
SRR505885	Calls	3141	3439	2253	2520	2628	4187
	True-positives	2541	2624	1719	2037	2161	**2712**
	Sensitivity	68.7	71.0	46.5	55.1	58.4	**73.3**
	Precision	**80.7**	76.2	76.2	80.8	82.2	64.8
	F1-score	**74.2**	73.4	57.7	65.5	68.3	68.8

**Fig 4:**
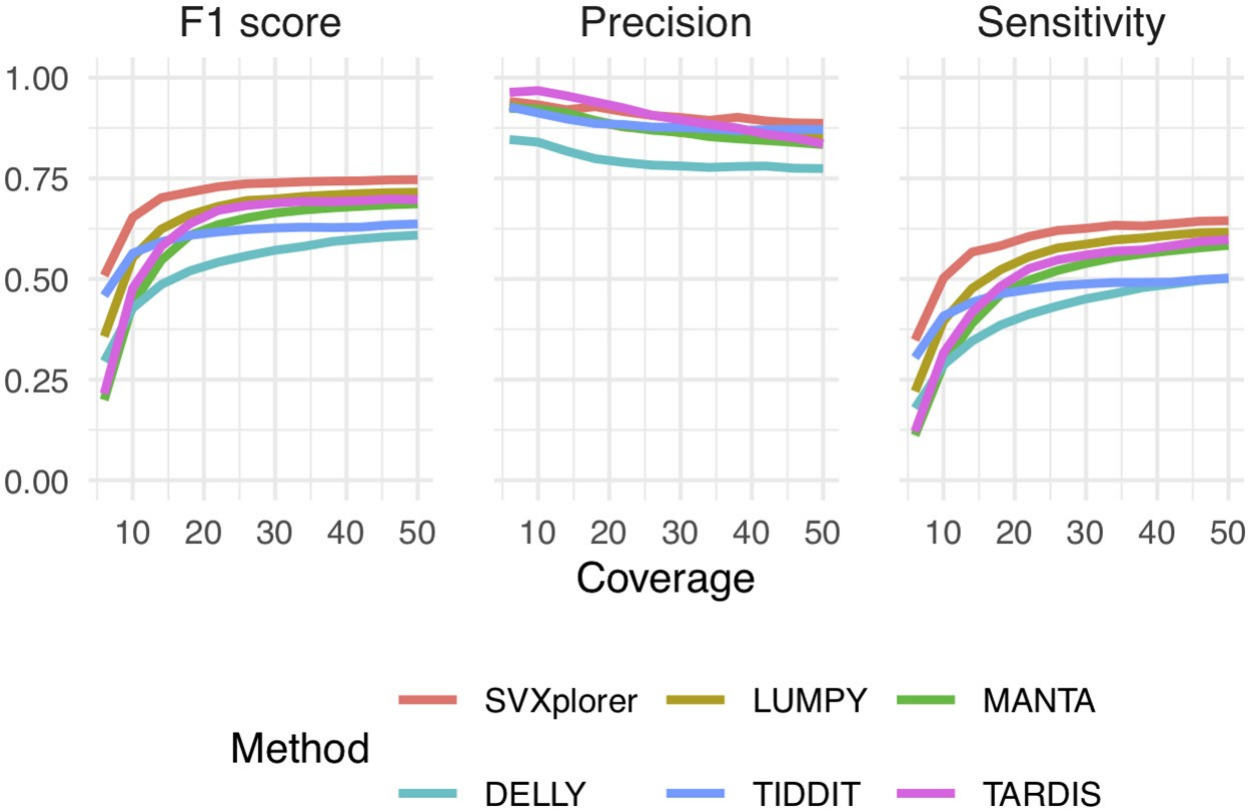
Sensitivity and precision vs coverage for ERR194147.

In addition, performance curves for sensitivity and precision with varying coverage were generated for all callers for the ERR194147 library against the PacBio deletion truth set. SVXplorer shows the highest sensitivity and precision even at lower coverage compared to the other callers (Fig 4). The performance curves for SRR505885 are shown in S13 Fig. SVXplorer has the highest F1 score compared to the other tools included in this study at all coverages that were sampled. We again show the distribution of sizes of deletion detected by SVXplorer for the ERR194147 library in S14 Fig.

Even though each library and subsequent short-read sequencing represents an independent experiment, it was natural to assess the concordance of the callers for the two experiments, considering that the data is from the same sample. We asked the question: “What percentage of calls made by each caller for one library were *found* in the other library”? We acknowledge that the results from such a comparison are fraught with caveats, and should not be considered in isolation. For example, a caller could repeat a systematic error and detect a variant in both experiments resulting in higher concordance. Alternatively, it could call a variant resourcefully by using sparse information in one experiment but miss it altogether in the other due to sparser information, resulting in an unfair penalty. However, we believe that this assessment provides an additional metric beyond sensitivity, precision and F1 scores that can be used to understand the differences between the various methods. Many different approaches can be used to evaluate this “reproducibility” between the experiments, and we have chosen a liberal approach to assess it in this study.

We take the final call-set of one library (called the “base library”) and compute its overlap with a call set including all unfiltered and ungenotyped SVs for the other library (termed the “liberal library”), allowing a slop of 200 bps between the detected breakpoints. For all tools, the final call set comprises of variants that passed all filters, and whose genotype was not homozygous/unknown for the reference if genotyping was implemented. The liberal library for all tools comprises calls either not labelled as “PASS” or with a genotype string of “0/0” or “./.” This is because we are interested in knowing whether a variant called in one sample presents reasonable evidence of being seen in the other sample, given various discrepancies and artifacts in fragmentation, sequencing and alignment. We perform the test with the caveat that the different callers may not be directly compared with each other, as the cluster support thresholds, mapping thresholds, the sheer volume of unfiltered calls, and clustering algorithms are all very different across the various callers. LUMPY, MANTA and DELLY all identify 2-breakpoint variants only, i.e., an “FR” cluster becomes a deletion candidate and an “RF” cluster becomes a duplication candidate. This is true for simple deletions and tandem duplications, but not when clusters with these signatures arrive from cut- or copy-paste insertions. To at least have the same framework as the other callers, SVXplorer’s self-consistency comparison is done at the cluster level (prior to integrated-variant formation). Essentially, all its PE and SR clusters that pass filters in the base library are compared to all PE and SR clusters in the other library (with “FR” clusters or equivalents termed “deletions” and “RF” clusters or equivalents termed “duplications” for uniformity across tools).

In other words, the collection of all 2-breakpoint clusters of any read orientation arising from PE reads, and all 2-breakpoint clusters arising from split reads are considered for comparison in each library. Clusters in the base library must belong to variants that passed disjointness and read-depth filters in subsequent stages. This collection of clusters is searched for in the liberal collection of the other library containing all unfiltered clusters. A base cluster must match read orientation and overlap within breakpoint slop with another cluster in the liberal collection to be counted as concordant. SVXplorer uses an intermediate file in its workspace called “variants.pe_sr.unfiltered.bedpe” to create the liberal cluster collection. BND events were not assessed in this comparison due to the potential uncertainty in the nature of such events, and hence in their reproducibility. Naturally, due to these differing frameworks, TIDDIT and TARDIS (the other two callers capturing 3-breakpoint events) could not be included in this self-consistency analysis. Since cluster-level information is not available for these tools to the authors it is not possible to provide them a similar framework as the other callers. For example, TIDDIT uses BND events to report adjacencies arising from its 3-breakpoint-variant calls. No tag is provided to separate these BND events from BND events unrelated to 3-breakpoint variants, thus rendering the task of extracting a set of clusters for TIDDIT similar to that of the other tools impossible.

**Fig 5:**
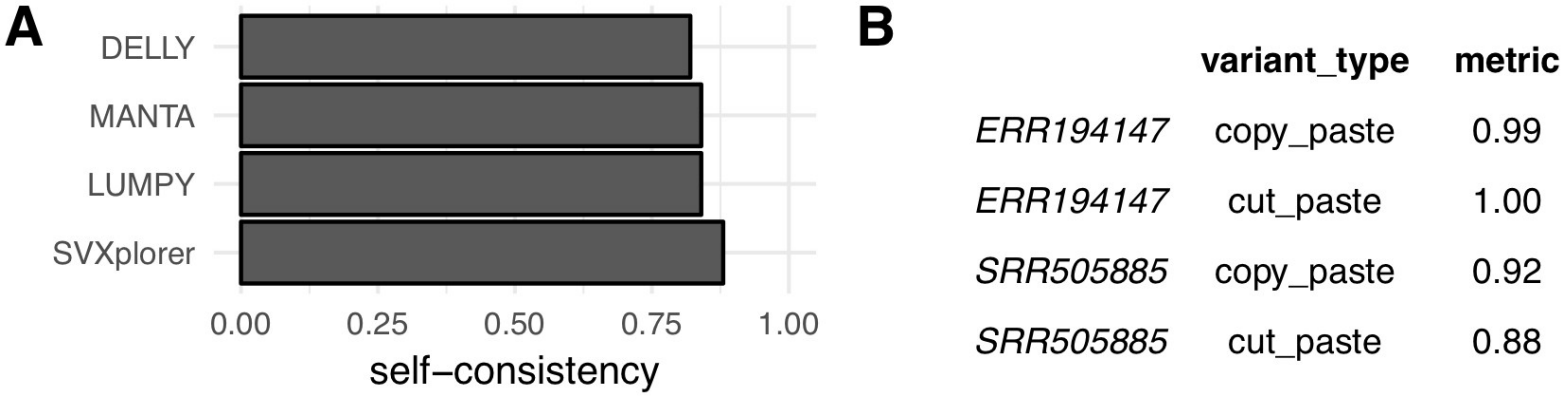
Self-consistency for NA12878. (A) Normalized self-consistency comparison for the various approaches. This quantity is obtained by weighing the SV-type self-consistency given in S15 Fig by number of each SV type. (B) Integrated-variant self-consistency for SVXplorer.

Fig 5A shows the overall normalized self consistency for the four callers. We show plots for each of the three common SV types along with calls by category for each caller in S15 Fig. All callers perform reasonably, as expected. SVXplorer shows high overall consistency in all categories, though of course, the callers are not directly comparable. As alluded to before, the average number of inversions called for the two libraries was 50 for SVXplorer, 30 for LUMPY, 350 for MANTA and 599 for DELLY. SVXplorer and LUMPY are much more in line with expectation compared to DELLY and MANTA for the called inversions [16].

We also evaluated the integrated-variant self-consistency for 3-breakpoint variants (cut- and copy-paste insertions) for NA12878 using SVXplorer and we report it in Fig 5B. The 2-breakpoint source location of all insertion calls made by SVXplorer (cut-paste and copy-paste) was extracted for one library and checked for simple overlap with an “FR” or “RF” cluster, or an integrated-variant source location of the unfiltered call set of the other library (see subsection on “NA12878 integrated-variant self-consistency” in the present section in S1 File for details). This check simply supports the correctness of the observed breakpoints of the 3-breakpoint variant for a given library via evidence of similar breakpoints seen for the other library. The overlap rate being very close to 100% in most cases substantiates that the variants are not products of artefacts in data but real SVs.

#### SVXplorer exhibits high self-consistency in a trio setting

We next evaluated the performance of SVXplorer on the data from the AJ trio sequenced as part of the Genome in a Bottle (GIAB) effort. We downloaded the fastq files for samples HG002, HG003, HG004 from ftp://ftp-trace.ncbi.nlm.nih.gov/giab/ftp/data/AshkenazimTrio. The reads were aligned against the b37+decoy human reference using BWA to a coverage of 25*X* coverage per sample. In general, trio analysis is also useful in testing result reproducibility and accuracy, i.e., we expect that all variants in the child should also be found in the parents and that there must be more variants shared between the child and one of the parents as compared to those shared between both parents. Noting that now we have different related individuals for assessing reproducibility instead of different experiments on the same individual, self-consistency was evaluated identically to NA12878 with the same caveats for all tools. The child’s final calls are treated as analagous to the final call-set of the base library and each unfiltered/ungenotyped call-set of the 2 parents is analogous to the call set of the liberal library. AJ trio self-consistency for the various callers is shown in Table 3. SVXplorer again has clear, high consistency in every category in this analysis—in terms of difference between calls shared between parents and those between child and either parent, in terms of calls found in child but not in either parent, and in terms of raw overlap of calls between child and parent. Please refer to the present section in S1 File and S16 Fig for more details.

**Table 3:** AJ trio self-consistency.

SV type	Approach	Calls	C-F	C-M	Not in M/F
Deletion	SVXplorer	2483	**0.76**	**0.79**	**0.036**
	LUMPY	2448	0.75	0.77	0.046
	MANTA	2807	0.68	0.70	0.115
	DELLY	1312	0.68	0.69	0.097
Duplication	SVXplorer	320	**0.69**	**0.7**	**0.103**
	LUMPY	315	0.64	0.66	0.146
	MANTA	234	0.62	0.58	0.184
	DELLY	495	0.62	0.59	0.186
Inversion	SVXplorer	46	**0.76**	**0.78**	**0.065**
	LUMPY	35	0.74	0.74	0.114
	MANTA	292	0.62	0.66	0.151
	DELLY	578	0.71	0.71	0.118

“C-F” refers to the overlap between calls of child and father for each SV category as a fraction of child’s total calls (first column), “C-M” to the same between child and mother, and the last column shows the fraction of the child’s calls that were not seen in either parent. The “best” result for each metric is highlighted in bold.

We also evaluated the deletion calls for HG002 against an available truth set generated using an ensemble approach in Parliament [24], and show the results from that comparison in Table 4. SVXplorer consistently outperforms the other callers in sensitivity, precision and F1 score. This certainly lends further credence to the substantiality of SVXplorer’s self-consistency results above. As a note, the standard deviation in insert length distribution is greater than 400 base pairs for all three data-sets.

**Table 4:** Performance of deletion calls for HG002.

	SVXplorer	LUMPY	DELLY	MANTA	TIDDIT	TARDIS
Calls	2478	2448	1312	2807	1958	1175
True-positives	**2057**	1969	795	2051	1605	940
Sensitivity	**61.2**	58.6	23.7	61.06	47.8	28.0
Precision	**83.2**	80.4	60.6	73.12	82.0	80.0
F1-score	**70.6**	67.8	34.0	66.54	60.4	41.5

The “best” result for each metric is highlighted in bold

#### Benchmarking of 3-breakpoint variants

We detected 185 cut- and copy-paste insertions in the child that passed all filters. Of those, 174 were non-inverted copy-paste insertions, 9 were cut-paste insertions with confirmed breakpoint order, and 2 were inverted copy-paste insertions. We used this dataset to evaluate SVXplorer’s performance on these insertion calls. We compared the call made for the child to calls made for each parent, and to a callset that contained the union of the calls from both parents. A true positive was registered when all breakpoints and the exact SV type of the child’s insertion call overlapped and agreed with the SV type of a call in the parent callset within a slop of 300 bps. A false negative was called if a call in the parent callset did not meet the above criteria. All calls in the child’s callset that were not true positives were recorded as false positives.

The parents’ callsets consisted of all 3-breakpoint variant calls tagged as cut-paste insertions with known breakpoint order or as copy-paste insertions that passed all filters. The results of this assessment are shown in Table 5. SVXplorer achieves about 45% or greater precision in comparisons against one parent, and close to 50% sensitivity in this evaluation. This is significant, given the novelty of such an attempt to benchmark 3-breakpoint variants, the low coverage of the AJ Trio dataset and the large insert-length spread seen in these data. The precision of the calls against the combined data further increases to 72% if we include BND calls from the parents that are tagged as most likely being cut-paste or copy-paste insertions (a “PROBTYPE” field in the VCF is listed for BND events). Please see Supplementary Results in S1 File for more details on this analysis.

**Table 5:** SVXplorer’s performance of 3-breakpoint variant calls.

	Total calls	Child Calls	Overlap	Sensitivity	Precision
Father	183	185	90	49.2	48.6
Mother	184	185	83	45.1	44.9
Combined	367	185	118	46.0	63.8

The child’s cut and copy insertion calls are evaluated against the parents’ in 3 different assessments. Overlap is defined as the number of calls in child where all 3 insertion breakpoints agree with a call in the parent call-set within a slop of 300 base pairs and share exactly the same SV type. Sensitivity and precision are evaluated for the child with the parent call-set acting as ground truth.

## Discussion

We have developed a structural variant caller that shows improvement over existing approaches on simulated variants and real datasets (haploid and diploid samples). It produces more consistent calls for related individuals as well as for different libraries for the same individual, compared to several other callers. It outperforms compared callers in precision as well as sensitivity, particularly when the coverage is lower or the insert length distribution is variable. Unlike most other SV callers, SVXplorer registers deletions and duplications arising from 3-breakpoint variants like translocations and copy-paste insertions, improving the precision of CNVs in the process.

Table 6 shows a comparison of the total user time and peak memory usage for the callers used in this study for three representative datasets. Among the methods that are capable of detection of 3-breakpoint events, SVXplorer uses memory comparable to TIDDIT and is significantly faster compared to both TIDDIT and TARDIS. There are several reasons for SVXplorer’s overall effectiveness and better performance. One significant reason is its probabilistic cluster formation algorithm that uses the insert length distribution via a precise, global binning to characterize the likelihood of two fragments belonging to the same variant in the sample, and is based on both the insert length difference and the alignment distance between the fragments. Another significant reason for better performance is its progressive recombination of clusters with specified signatures to form two- and three-breakpoint integrated variants, which does not call individual clusters as variants until it has exhausted all predefined possibilities of signature matches. Most callers that rely on paired-end signatures annotate RF clusters as evidence for a duplication and FR clusters as evidence of deletion. This can be tricky, just as one example, in the case of breakpoints generated by retrotransposons that can ‘copy and paste’ their genetic code around the genome. The signature of such calls from discordant reads is the overlap of 1 RF and 1 FR cluster. Without cluster consolidation, a putative deletion and duplication in the region would be marked with incorrect breakpoints (Fig 1). If read-depth filters are reliable, no variant would be called in the region. Although a robust truth set for duplications and translocations for real data was not found, we showed that even in simulations sensitivity was significantly compromised for other callers in such regions. And if the read-depth filters were less reliable (as is the case in real data) and the putative deletion or duplication were called with incorrect breakpoints, the sensitivity and precision of calls arising in these regions would both decrease.

**Table 6:** Total CPU time and peak memory usage for the various methods.

Dataset	Method	CPU time (minutes)	Peak memory usage (GB)
Simulation (50X)	SVXplorer	25.20	1.25
	MANTA	91.58	0.10
	LUMPY	9.91	1.07
	DELLY	160.57	0.30
	TIDDIT	106.60	3.57
	TARDIS	108.59	8.72
ERR1341794 (40X)	SVXplorer	63.12	3.36
	MANTA	425.63	0.26
	LUMPY	159.55	3.10
	DELLY	839.45	1.34
	TIDDIT	123.34	3.59
	TARDIS	157.16	9.45
CHM1 (40X)	SVXplorer	80.98	4.43
	MANTA	833.26	0.21
	LUMPY	193.59	5.28
	DELLY	766.44	0.99
	TIDDIT	142.12	3.59
	TARDIS	891.72	12.07

For SVXplorer, if reads aligning to the “copy” (source) and “paste” locations have reasonably high mapping quality (a minimum of 20 is required for both sides of an RF alignment-pair), the concern of these alignments coming from repeated genomic regions is much less. Even if the source region of the insertion had low mappability, the question of “where” the insertion came from is a moot one in the presence of such identical regions in the genome. Further, of similar clusters, the largest one containing the most number of continuous alignments is typically picked by SVXplorer. The efficacy of such insertion calls by SVXplorer has been demonstrated as best as possible using repeat bias in simulated insertions, self-consistency of clusters comprising insertions assessed in two different sequencing libraries, and via its integrated-variant self-consistency. SVXplorer’s comprehensive consolidation for insertions arising through ‘cut and paste’ and ‘copy and paste’ mechanisms, inversions, and even tandem duplications enhance its putative call set by reducing false positives among deletions and tandem duplications while identifying accurate, complete insertion sites. Both PE and SR alignments are used individually and collectively to exhaustively form all putative integrated variants with specific signatures. For example, a cut-paste insertion requires at least 3 supporting clusters of specified signature (S4 Fig), which may be read in any order, containing either inverted or non-inverted alignments, and which therefore need to be carefully handled to finally form the 3-breakpoint cut-paste variant. All support thresholds (which are dynamic and coverage-based) and filtering are applied not to individual clusters but to integrated variant blocks. Read-depth filtering is applied to streamline these calls further with enhanced features, such as the use of only mappable regions to calculate local coverage and ignoring regions deemed otherwise unreliable in the variant. Zero-mapping-quality alignments are also systematically ignored in read-depth analysis.

SVXplorer exhibits an increase in sensitivity and precision in comparison to the other approaches for multiple datasets, even though the improvement can appear marginal in some cases. But even if the improvement is marginal in terms of metrics such as sensitivity, the differences between the methods based on breakpoints and their overlaps can be significant. For example in the CHM1 dataset, only 1597 of the 1759 deletion calls made by SVXplorer overlapped calls made by LUMPY. Of the 162 calls (10%) unique to SVXplorer, 101 of them overlap the truth set within a slop of 200 bps. Similarly, for LUMPY, 41 of the 207 calls unique to it are true-positives using a slop of 200 bps. Several enhancements to SVXplorer (and this line of research) can be envisioned that would improve its utility and performance. First, SVXplorer does not have an explicit mechanism to identify insertions and deletions smaller than both the insert-length standard deviation and the lowest primary alignment length for the SV. Another area of improvement for SVXplorer is in the handling of multi-allelic variants. For example, a deletion and a duplication with similar reference breakpoints may not be called by SVXplorer as it could be annotated as a copy-number invariant region in the final filter. Such variants, however, can be identified in a family trio by post-processing the identified variants. The current version of SVXplorer does not model biases in sequencing, relying on a careful examination of read-depth instead. However future versions should be able to incorporate better models of read-depth using single-position models, speeding up the execution of the approach further.

Future research can also build on SVXplorer’s methodology to precisely model 3-breakpoint variant signatures stemming from copy-paste and cut-paste insertions, and inverted transcriptions, in real data presenting complicated alignments and possibly confounding signatures. Simulations of the human genome with such artificially inserted events can be done to corroborate these models. Several callers could then come up with ensemble truth sets for human non-tandem duplications, which are quite sparse at the moment. One could also characterize more precisely the nature, frequency, size distribution etc. of these duplications and translocations in a healthy genome, and progressively use these findings to aid in classification of diseases associated with atypical genomic rearrangements.

## Methods

SVXplorer requires a coordinate-sorted BAM file generated by aligning Illumina paired-end reads belonging to a single read-group against a reference genome as input. It calculates the coverage and insert length distributions from this BAM file, and groups the fragments that are marked as discordant by the aligner into sets we refer to as clusters. All fragments in a cluster are required to have the same relative orientation of their constituent reads after alignment, and are selected so as to support the same putative variant. It then tests if the clusters can be further grouped into integrated variants such as inversions and translocations based on breakpoint overlap and their combined signature. Split-read evidence from the BAM is then incorporated, both to support existing variants and to create variants that were not captured using the discordant paired-end reads. SVXplorer then processes the variants to remove calls that could be caused due to errors in sequencing or alignment. Finally, read-depth information is added to all the variants and used to further filter the set of calls. In case of multiple read-groups as observed in several datasets, SVXplorer should be run on each one of them separately. We now describe each of these steps in detail.

For clarity, we first define a few terms that are used in the subsequent sections. We refer to the DNA insert as used in Illumina sequencing as ‘fragment’ (S1 Fig). The “tip” or “head” of an alignment refers to the largest genomic coordinate in case of an alignment to the forward (F) strand, and the smallest genomic coordinate in case of an alignment to the reverse (R) strand of the reference genome. The “tail” analogously refers to the smallest genomic coordinate of a forward-oriented alignment and the largest coordinate for a reverse-stranded alignment. An FR alignment thus would refer to an alignment whose left mate is forward-oriented and whose right mate is reverse-oriented on the same chromosome. “Mappable” regions refer to regions in the reference that are unlikely to contain reads with poor mapping quality and were identified by running GEM mappability [27] on the reference genome. A “small” cluster refers to a discordant PE cluster that is composed of discordant alignments where the observed insert length is smaller than the estimated mean insert length. A “variant map” refers to the set of all relevant supporting fragments of a putative variant. An “integrated” variant is any variant composed of more than one discordant alignment cluster or one giving rise to more than one discordant alignment cluster. SVXplorer integrates clusters arising from cut- and copy- paste insertions, inversions and in some cases tandem duplications into these respective variants. A “3-breakpoint variant (event)” refers to a cut-paste or a copy-paste insertion event, which has 2 breakpoints spanning the source of the insertion and 1 identifying the target location where it is pasted in the genome. A “breakpoint region” is the combination of all locations in the reference where the true breakpoint is estimated to possibly exist. A variant whose support tag is “mixed” has support from both PE and SR alignments.

### Preprocessing

In this step, we subsample alignments from the input position-sorted BAM file to calculate the insert length and coverage distributions in the dataset. We filter the BAM file to keep discordant reads that pass preset thresholds relative to the mean of the insert lengths and respective mapping quality thresholds. These are then used as input to the next step (see section “Preprocessing” in Supplementary Methods in S1 File for details).

### Formation of paired-end clusters

We group fragments aligning discordantly into “clusters” that have the same relative orientation of the reads, and putatively support the same structural variant. Briefly, each fragment with a discordant primary alignment is taken as a node in a graph *G*, and an edge is created between two nodes *i* and *j* if and only if a calculated score *W*_*ij*_ for the pair exceeds a predefined threshold. After all the node pairs in a genomic region have been investigated, connected components from the graph are identified and the nodes in each connected component are separated into maximal cliques using a greedy set-cover approach. S2 Fig shows the size-distribution of connected components in one such dataset. Each clique is treated as a set, and the maximum clique (or largest maximal clique) in the collection of cliques, is processed into a cluster, i.e., its member fragments are used to determine the cluster’s breakpoints and error margins. Once a clique is processed, all its member fragments are removed from all other sets, and are not used as part of any other cluster. The clique set itself is now removed from the collection of cliques in the connected component and the steps are repeated. All cliques that have members fewer than a predefined threshold are ignored. All fragments that are part of the same clique are mutually proximal (per the weight criteria) and purportedly support the same variant. The idea of using cliques to group alignments into clusters is not new, and has been used by CLEVER [17], VariationHunter [28] and others. SVXplorer, however, calculates the edge-weights starting with a first-principles probabilistic approach and uses the insert length distribution in two significant ways with precision which leads to improvement in performance compared to other tools that use similar approaches. Please refer to the present section in Supplementary Methods in S1 File for further details.

In order to motivate how the score *W*_*ij*_ is calculated, we now present a heuristic argument. Let us define *C*_*ij*_ as the event that two aligned fragments *i* and *j* drawn at random from the genome support the same variant. *The connection weight W_ij_ is a calculated score for the probability of the event C*_*ij*_. The distance profile of a pair of fragments *i* and *j*, *D*_*ij*_, is a function of the difference of the insert length of the two fragments accounting for the orientation of the reads in the alignment and the distance between the respective left reads of the fragments. We denote the observed *difference* in the insert length between the two aligned fragments as Δ_*ij*_ and the observed “tip-to-tip” distance between respective left alignments as *L*_*ij*_. Using Bayes’ rule, *X*_*ij*_ = *P*(*C*_*ij*_|*D*_*ij*_ = *d*_*ij*_) is given by:

$$X_{ij}=P\left(D_{ij}=d_{ij}|C_{ij}\right)\cdot \frac{P(C_{ij})}{P(D_{ij}=d_{ij})}$$ (1)

$$=P\left(D_{ij}=d_{ij}|C_{ij}\right)\cdot \frac{P(C_{ij})}{P\left(D_{ij}=d_{ij}|C_{ij}\right)\cdot P\left(C_{ij}\right)+P\left(D_{ij}=d_{ij}|C_{ij}^{c}\right)\cdot P\left(C_{ij}^{c}\right)}$$ (2)

where $C_{ij}^{c}$ denotes the complement of the event *C*_*ij*_. We take note here that the overall probability *P*(*C*_*ij*_) does not depend on the distance profile, whereas the other terms in (2) do. We would also like to point out that *P*(*D*_*ij*_ = *d*_*ij*_|*C*_*ij*_) is typically a monotonically decreasing function of Δ_*ij*_ and *L*_*ij*_, and $P(D_{ij}=d_{ij}|C_{ij}^{c})$ is typically a monotonically increasing function of the same two quantities. The event $C_{ij}^{c}$, among other things, includes the possibilities that the fragments belong to different variants or are sampled from systematic misalignments that resemble true variants. Assuming an unimodal insert length distribution and given that alignments clustering together in the reference arising from true variants far outnumber systematic misalignments that cluster together, the monotonic behavior cited above should be obvious. In other words, as the *difference* in insert length between two different fragments with discordant alignments rises, the likelihood of their being sampled from the same genomic region decreases. Further, as the distance between the respective read alignments on either side (e.g., left reads) rises, the likelihood of their belonging to the same variant cluster decreases. It may be more apparent now from (2) that *X*_*ij*_ is a monotonically decreasing function of Δ_*ij*_ and *L*_*ij*_, as the term multiplying *P*(*C*_*ij*_) is always less than 1. Also, the only term in (1) that is grossly dependent on the distance profile is *P*(*D*_*ij*_ = *d*_*ij*_|*C*_*ij*_). The denominator in (1) is also dependent but, given the spread/smattering of discordant alignments in the genome, it has opposite monotonicity to the numerator and only supports the same monotonic behavior. Thus, it need not be further treated or considered for this heuristic motivation of the connection weight. Since the algorithmic objective is to define a fragment-connection weight that is monotonically and structurally similar to *X*_*ij*_, the following function, a practical reproduction of *P*(*D*_*ij*_ = *d*_*ij*_|*C*_*ij*_), is chosen to define the score between two nodes *i* and *j*:

$$\begin{array}{c} W_{ij}=\theta_{ij}\cdot P\left(\Delta =\Delta_{ij}|C_{ij}\right)\cdot T\left(L_{ij}\right)\text{,} \end{array}$$

where *P*(Δ = Δ_*ij*_|*C*_*ij*_) is directly obtained from the subsampled insert length distribution by binning the insert length difference values, and taking the ratio of the number of entries in the bin in which Δ (the observed insert length difference) resides to the total number of entries. *T*(*L*_*ij*_) is a function that penalizes distance between the respective left alignment reads after the distance crosses a certain threshold. The penalty threshold for *T*(*L*_*ij*_) is chosen to be the “generalized 3 sigma” (*σ*_3_) mark, which is the insert length value at the 99.85 percentile mark (which is equivalent to the 3-sigma mark for Gaussian distributions) of the insert length distribution. The penalty is a simple linear cost that takes $T(d_{0_{ij}})$ to 0 at *p*_*mi*_, the insert length at the 99.9999 percentile mark of the insert length distribution. Thus

$$\begin{array}{c} T\left(L_{ij}\right)=\{\begin{array}{cc} 1 & \text{if}\;L_{ij}<=\sigma_{3}\\ 0 & \text{if}\;L_{ij}>p_{mi}\\ 1-\frac{L_{ij}-\sigma_{3}}{p_{mi}-\sigma_{3}} & \text{otherwise} \end{array} \end{array}$$

*θ*_*ij*_ is an indicator variable that is 1 if the two fragments (a) have the same relative orientation of reads, and (b) align to the same set of chromosomes. If the relative orientation of the reads is “FR” then they are also required to agree on whether the insert length of the fragments is significantly higher or lower when compared to the average insert length. Currently, a suitable connection weight threshold is applied to the graph: *W*_*ij*_ > 0, i.e., all fragments that have a positive probability of being pairwise connected are connected to each other. However, the overall structure of *W*_*ij*_ is important, as in future work connection weights are envisioned to be edge weights in the graph *G*, and are to be used in generation of maximal weighted cliques. It is also an important consideration in the regime of low *P*(*C*_*ij*_), as the structure of *W*_*ij*_ includes hard cutoffs to 0 from discrete sampling of the insert length distribution. After all edges are formed, we find all the maximal cliques of each connected component [29] using an implementation from the NetworkX package [30]. The cliques are processed into clusters with breakpoints appropriately calculated according to the orientation of the reads. The breakpoint region size or breakpoint margin for each breakpoint of the cluster is given by:

$$\begin{array}{c} \sigma_{3}-\left(X_{R}-X_{L}\right)\text{,} \end{array}$$

where *X*_*R*_ is the location of the “tip” of the rightmost read supporting the breakpoint, and *X*_*L*_ the location of the “tip” of the leftmost read. Though SVXplorer lists precise variant breakpoints when split reads are present, using *σ*_3_ provides a conservative estimate of the breakpoint margin for calls supported only by PE alignments. Statistically, at most 2 variant calls out 1000, for any insert-length distribution, will have breakpoints lying outside the listed breakpoint region (for example when the observed alignments happen to begin at the edge of the true variant region and have the largest possible insert size per the insert-length distribution). Thus, the breakpoint margin is a conservative estimate even for insert length distribution of anomalous shapes such as those generated when enzyme-based fragmentation methods are used. Of course, for typically Gaussian distributions, *σ*_3_ happens to lie about 3 standard deviations from the mean. As SVXplorer’s edge-weight calculation depends on statistics from a given insert length probability distribution, its efficacy is independent of/takes into account the variance of the distribution.

So, simplistically, fragments are likely candidates for belonging to the same cluster if their mutual insert length difference and their mutual distance are both low. The mutual distance of aligned fragments being low is not implied by a mere overlap of the alignment regions if the left and right alignments are distant. The insert length distribution (ILD) in reference was the most natural candidate for efficient computation of this likelihood. SVXplorer strives to represent the likelihood of *C*_*ij*_ with as much useful precision as the available information allows and uses the ILD via a global binning approach to extract such data-based precision in fragment discrimination.

### Consolidation of paired-end clusters into variants

The clusters that are formed at the end of the previous step are tested for overlap with each other. Cluster “overlap” is defined by overlap of the breakpoint regions in a manner that the composite signature agrees with a specific type of integrated variant. Clusters that overlap are grouped and tagged as part of a putative integrated variant. In fact, each cluster is first compared to all such existing variants for possible matches and then to all clusters that are not yet integrated. This allows a variant to be composed of more than two clusters (e.g., as in translocations). Variant sets are then formed for all variants by union of respective member cluster sets, recording all the alignments that support a given variant.

Cluster consolidation is detail-intensive (for example, S5 Fig), and carefully performed for all basic structural variant (SV) categories that we currently consider. The well-known SV categories used are: deletion, tandem duplication, inversion, *de novo* (or non-reference) insertion, and other insertions that occur using a copy- or cut-and-paste mechanisms.

- Deletion (**DEL**): An “FR” cluster that has not been paired with any other cluster and where the included fragments have an insert length that is significantly larger than the average insert length.
- Tandem duplication (**TD**): An “RF” cluster that has not been paired with any other cluster.
- Inversion (**INV**): A pairing of 1 “FF” and 1 “RR” cluster due to the overlap of both left and right alignments respectively.
- Insertion resulting from a copy-paste mechanism (**INS**): A pairing of 1 “FR” and 1 “RF” cluster. An exact signature match as shown in the S3 Fig is required.
- Insertion resulting from a cut-paste mechanism (**INS_C**): A pairing of 1 “FR” and 1 “RF” cluster as above, but another “FR” deletion cluster flanking 2 adjacent breakpoints (S4 Fig). If all 3 breakpoints lie on the same chromosome (indicating an intrachromosomal translocation), this is a symmetric situation in the 3 breakpoints and it is not possible to distinguish the source of the translocation from the location where it is pasted without using read-depth information. If identified, the paste location breakpoint is defined as “1” and the source locations are defined as “2” and “3”, and the variant is labelled **INS_C_P**.
- De novo insertion (**DN_INS**): A pairing of clusters that are composed of alignments with only one mapped mate and whose alignments have mutually opposite orientation, or an unmatched small “FR” cluster indicating a (novel) inserted segment between its left and right breakpoints.

SVXplorer allows for a detailed treatment of SV types and categories not typically identified using other approaches. Please refer to the present section in the Supplementary Methods in S1 File for a more detailed explanation of these signatures.

### Incorporation of split-reads

In this stage, split reads are both used to add support to existing variants and form new variants. Split read alignments (extracted using extractSplitReads_BwaMem script included with LUMPY [6]) are compared to all existing putative variants they could support. If an SR alignment supports a given PE variant call with the correct signature, the variant support tag will now include “SR” and the supporting fragment will be added to the variant map of said variant (see S7 Fig and “Incorporation of split-reads” in Supplementary Methods in S1 File). If the split alignment does not match any existing (PE or SR) variant, then it is stored as a new possible SR variant. As with PE calls, this new SR variant can be composed of/consolidated by different read signatures, and can be a 2-breakpoint or 3-breakpoint variant.

Variant categories that are created based on SR evidence with no evidence from PE reads are: deletion/insertion, tandem duplication/insertion, insertion and inversion. A brief description of these signatures is provided now, and we include a detailed explanation in the Supplementary Methods in S1 File in the cited section.

- Deletion/insertion (**DEL_INS**): A split read yielding unswapped (please refer to Supplementary Methods in S1 File for detailed explanation of swapping) “FF” or “RR” alignments on the same chromosome is marked as a deletion/insertion candidate. Such a cluster can be supported by both “FF” and “RR” split reads. If this cluster later matches with another cluster, giving rise to a third breakpoint, then it is promoted to an insertion (see S6 Fig). Insertions can be inverted or non-inverted. Depth of coverage is used to disambiguate these calls at a later stage.
- Tandem duplication/insertion (**TD_I**): A split read with the same orientation on the same chromosome that is a swapped read is marked as a tandem duplication/insertion candidate (S8 Fig). Again, it can be promoted to purely an insertion as in the case above. Depth of coverage is later used to disambiguate these cases where possible.
- Insertion (**INS**): Any split read whose segments map to different chromosomes is an insertion candidate. To be counted as a complete insertion, it must match with split reads that create a third breakpoint via the mechanism described above (S6 Fig).
- Inversion (**INV**): A split read yielding alignments with opposite orientation on the same chromosome is an inversion candidate. To be counted as a complete inversion, an inversion candidate cluster must match with another containing alignments which join the other side of the inversion to the reference. Merely oppositely oriented clusters are not sufficient. See present section in Supplementary Methods in S1 File for more details.

### Variant filtering

The following was conceived as an improvement on most SV-calling methods using fixed support thresholds to filter variants. SVXplorer dynamically calculates its support threshold, which is determined empirically by using a linear model based on the coverage of the dataset. The model assigns a support threshold for any given coverage in question based on linear interpolation of a plot of F1 score against coverage for simply simulated data containing common SV types (please see Methods in S1 File for more details). The support threshold set (PE, SR, mixed) for coverage = 25*X*, for example, was (4,4,4). This empirical threshold is now required to pass another filter.

All the variant sets formed thus far can either be completely disjoint or overlap with other variant sets, i.e., share clusters. We require that the *disjointness* of a variant set be the deciding factor in its inclusion in the final stage of processing. For any given data set, if a variant has at least as many supporting alignments (above the mapping quality threshold) as determined by the dynamic coverage model above, and if these are not shared by any other set, then the variant passes the disjointness filter and is processed further (please see S9 Fig for additional details). For robustness, the statistical procedure used to call variants here is applied to the complete variant set, not to individual clusters. This procedure was initially conceived for secondary alignments, but works just as effectively if only primary alignments are used. It was motivated by a set-cover algorithm based on disjointness which we present in Supplementary Algorithms in S1 File.

### Incorporation of depth of coverage

This stage carefully evaluates all the variant calls using local coverage and filters. Local coverage values in regions between reported variant breakpoints are investigated and if the average coverage in the region seems to contradict the variant in question, then the variant is written as a breakend (BND) event. A BED file listing all mappable regions is recommended as input from the command line and is used to identify regions whose local coverage values can be used in filtering.

In order to calculate variant-region coverage, sampling of bases is done from the middle and edges of the variant region, and only if absolutely necessary, with caveats, from the breakpoint margins. This coverage is assessed relative to the coverage for the chromosome and used to promote potential SR calls to putative variants, or reject PE calls as putative variants. The preset thresholds for deletion and duplication are chosen to be.8 and 1.2 respectively, which is the general standard for typical coverage distributions (due to the presence of other checks and balances they stand even if seemingly liberal for other cases like enzymatic fragmentation). If the ratio of average local coverage in the deletion/duplication variant to the chromosomal median coverage (variant coverage ratio, or *VCR*) exceeds/drops below its respective threshold, then in special cases such variants are not recorded.

This stage uses only mappable bases for assessing contig coverage as well as to assess local variant-region coverage as far as possible. Variant calls from all types of clusters (PE, SR, mixed) are rejected if sufficient number of bases (mappable or otherwise) did not exist to calculate coverage in the variant region. Further, coverage is also used break the symmetry of the 3 breakpoints for intrachromosomal translocations and corroborate the source (“cut”) and destination (“paste”) breakpoints. Please refer to the present section in Supplementary Methods in S1 File for more details.

## Supporting information

S1 File Supplementary methods, results and algorithms. (PDF)

S1 Fig Fragment and read as defined for this study. (PDF)

S2 Fig Distribution of size of connected components in ERR194147. (PDF)

S3 Fig A simple copy-paste insertion. The segment in orange is duplicated downstream in the sample. The figure shows 2 distinct clusters in red and blue matching up in the reference to form a copy-paste insertion. Breakpoint 1 (*x*_1_) is defined to be the overlap of adjacent oppositely-oriented alignments from the 2 clusters, and breakpoints 2 and 3 (*x*_2_ and *x*_3_) are defined by their respective mate alignments, with *x*_2_ < *x*_3_ by convention, whether upstream or downstream from *x*_1_.
(PDF)

S4 Fig A simple cut-paste insertion (translocation). The segment in orange is deleted and pasted downstream in the sample. The figure shows 3 distinct clusters, shown in red, blue and light orange. The cluster shown in light orange is the extra “FR” cluster resulting from the deletion of the translocated segment.
(PDF)

S5 Fig A “crossover” TD cluster. The segment in yellow is adjacently duplicated downstream in the sample. The figure thus shows sequenced fragments from a tandem duplication that align as “FR.” In such a case, the left breakpoint is defined by reverse alignments and the right breakpoint is defined by forward alignments.
(PDF)

S6 Fig A copy-paste insertion call from split reads. The segment in yellow is duplicated downstream in the sample. The orange read by itself would lead to a TD_I call and the blue by itself to a DEL_INS call. But together they define a copy-paste insertion consisting of 3 distinct breakpoints.
(PDF)

S7 Fig Example of a PE deletion call supported by a split read. The read shown in yellow (size exaggerated in target) is split into 2 alignments in the reference close to the PE breakpoints. The segment in green is the putative PE deletion call and the segment in yellow shows revised precise breakpoints.
(PDF)

S8 Fig A TD_I call from split reads. The segment in orange is tandem-duplicated downstream in the sample. The read shown in orange splits in alignment at the point shown in blue. The split partners are swapped in alignment, i.e., the head portion of the original forward-oriented read aligns in the reference to the left of the tail portion of that read. Such cases give rise to a TD_I cluster.
(PDF)

S9 Fig A special case. A case where 2 PE clusters each separately match up with a third cluster. The clusters in red and green match up with each other and so do the ones in red and blue, each matching pair indicating a copy-paste insertion. It is quite unlikely that both are true. This is addressed in the filtering stage.
(PDF)

S10 Fig Precision vs coverage for simulated data. (PDF)

S11 Fig Performance of SVXplorer compared to TIDDIT in detection of 3-breakpoint events. (A) Sensitivity with varying coverage (B) Precision with varying coverage.
(PDF)

S12 Fig Size distribution of deletions, duplications and inversions detected by SVXplorer for CHM1. (PDF)

S13 Fig Sensitivity and precision vs coverage for SRR505885. (PDF)

S14 Fig Size of deletions detected by SVXplorer and comparison with other methods for the ERR1341794 library. (PDF)

S15 Fig Self-consistency in NA12878 data when various approaches are used. “Consistency” refers to the fraction of calls in the listed base library that were found in the other library.
(PDF)

S16 Fig AJ trio self-consistency for the various SV types. “A-B” refers to fraction of total calls made for A that were found in B. Here A or B is a placeholder for either child, father or mother. “Difference” refers to the difference between fraction of calls in common between the parents and that between child and a parent (normalized). We expect this to be large. The “not found” column shows the fraction of total calls that were made in the child that were not found in either parent.
(PDF)
