# Supplementary material for: SVXplorer: Three-tier approach to identification of structural variants via sequential recombination of discordant cluster signatures
Source: PLoS Comput Biol. 2020 Mar 17;16(3):e1007737. doi: 10.1371/journal.pcbi.1007737 (PMC7100977; doi:10.1371/journal.pcbi.1007737)
Supplement: S1 File — (PDF) [file pcbi.1007737.s001.pdf]

1 Supplementary Methods

S1 Fig illustrates a fragment and a read as defined for Illumina sequences throughout this study.

1.1 Preprocessing

In the sample BAM, only fragments (a) that are marked as concordant by the aligner, (b) where both reads from the fragment align uniquely, and (c) the reads passing a preset alignment-score threshold are used to calculate insert length and coverage.

We filter the BAM file to keep discordant reads that pass preset insert length thresholds relative to the mean (both on $sigma_3$ as defined in Methods for both positive and negative deviations from the mean) and respective mapping quality thresholds. This preset mapping quality threshold is 1 for "FR" clusters and 20 for "RF" clusters, as the latter indicate duplications – which are fraught with surrounding repeats.

1.2 Formation of paired-end clusters

SVXplorer used the NetworkX implementation for forming connected components and maximal cliques using aligned fragments as nodes. The whole stage of paired-end cluster formation took 19 minutes for the ERR194147 library of the NA12878 sample at 50X coverage. A distribution of connected component sizes past the minimum cutoff of 2 fragments formed by the NetworkX implementation is given in S2 Fig for the interested reader.

As part of the cluster formation stage, fragments that seem highly aberrant based on relative left and right tip positions are removed from the cluster in question using a k-means approach. Alignments once finally written in a cluster are not used or counted in subsequent cliques, and clusters with size < 3 are not written by default.

It is worth mentioning here that a "cluster preservation" routine exists for paired-end clusters and can be activated by the user. The dynamic cluster-support cutoff for SVXplorer starts with a minimum requirement of 3 total supporting reads (PE+SR). Thus, the routine retains PE clusters with size < 3 at this point for subsequent stages. f split reads exist in the breakpoint regions of these clusters such that the combined (PE+SR) fragment support for the cluster is greater than 3, the cluster is preserved for subsequent stages. This increases run-time for high-coverage data sets but could improve results with low-coverage data.

Several structural variant callers identify regions of the genome where two or more clusters together imply conflicting calls. These conflicts are caused by 3 variants existing successively within each other, such as 2 deletions and a non-deletion, 2 inversions and a non-inversion etc. SVXplorer did not detect any conflicts of this nature. We impose a more stringent criterion and identify regions that contain multiple clusters with the same orientation close to each other. Such regions are typically indicative of misalignments. We store the coordinates for such clusters in a separate file, and these regions are subsequently processed to ascertain if any of the clusters can be selected based on a predefined threshold of proportion of alignments in the region it accounts for, and included in subsequent analyses.

Immediately after formation of PE clusters, a blacklist of "unclean" cluster regions is made by sweeping through all regions containing multiple clusters and marking those that present non-standard signatures for cluster overlap/consolidation. In other words, regions where multiple clusters exist but do not seem to combine to support one complex variant in that region are blacklisted integrated variant in that region are blacklisted (it is not so likely that two integrated variants would exist in the same region) One such example is the existence of 2 successive RF paired-end clusters with overlapping margins.

1.3 Consolidation of paired-end clusters into variants

SVXplorer combines signatures from all clusters laboriously, and updates the SV-label of existing consolidated clusters upon seeing new evidence. A copy-paste may get updated to a cut-paste, for

example, or a small-variant cluster (explained below) may be absorbed into a putative cut-paste variant without any change in type. These variants are also categorized by inverted or non-inverted paste mechanisms. Split-read calls are dealt with similarly.

As referenced in the manuscript, copy-paste and cut-paste insertions are shown and described in more detail in S3 Fig and S4 Fig.

The VCF file groups multiple events (DUP, BND) coming from one cut-paste or copy-paste insertion via the GROUPID subfield (in "INFO") in order to preserve all the information of the BEDPE output. It also contains a "PROBTYPE" subfield where the likely but undetermined SV type of a BND event (e.g. "Translocation") is printed. An "ISINV" flag is printed in the "INFO" field if a duplication is inverted.

SVXplorer attempts to account for many special cases of SV formation. One worth mentioning here is the "crossover" TD cluster. As shown in S5 Fig, such a cluster is formed when the insert length is comparable to the size of the tandem-duplicated segment. The cluster consists of paired-end alignments whose reads are aligned as "FR" (but are very close or may even overlap each other). Therefore, the left breakpoint of the cluster is defined by reverse-stranded alignments and the right breakpoint is defined by forward-stranded alignments. This is unlike a deletion "FR" cluster and is processed as a tandem duplication. It is also of note that a small "FR" discordant cluster can be formed around any of the above variant types that involve an inserted segment (i.e., tandem duplication and all insertions) if the insert length is comparable to the variant size. Thus, such a cluster can be part of any of the above signatures involving inserted segments.

1.4 Incorporation of split-reads

If the alignment signature supports an existing variant and the split alignments have breakpoints within existing breakpoint margins (see S7 Fig), then we use the read alignment in the reference to update the current variant breakpoints and tag the variant as "precise". The alignment record is added to the existing variant set in the variant map, and the variant is tagged as "PE_SR"-supported. For example, an "FF" PE cluster may now be supported by an "FR" SR cluster, and if it happens to join the reference on the other side of the potential inversion as the PE cluster, then it completes the putative inversion (there is a "liberal inversion" parameter that can be set by the user to merely require support for inversions by PE and SR reads to be called). In comparing alignments with existing SR variants a small bidirectional "slop" is used to account for possible imprecision in some reads (5 by default).

Variants called exclusively by split-reads are more limited in their scope compared to PE variants for Illumina reads. SR coverage is typically lower than PE coverage, which may render SR cluster matching and consolidation less reliable considering the narrower Poisson window for such reads in sequencing (reads overlapping variant boundaries, as opposed to the more numerous whole reads outside boundaries supporting the variant). For example, a single split read may be aligned such that its two split partners align with the same orientation ("FF" or "RR") in the reference. This could indicate a deletion or "two-thirds" of a copy-paste insertion or translocation (see S6 Fig). Thus, if an "FF" or "RR" split-read cluster is unmatched till the end, it is not labelled a putative deletion immediately but only if it passes stringent deletion filters in the final pile-up filtering stage as described below.

If the split partners are swapped, then they could indicate a tandem duplication or a copy-paste insertion/translocation. A *swapped* alignment is defined as a split read where one split partner sequence came before the other in the sequencing direction in the sample, but now comes after the other in the alignment direction in the reference (as in S8 Fig). The swap is determined by extracting the relative query start and end positions from the BAM file (applicable for all "FR" and "RF" alignments). New SR variant categories are: deletion/insertion, tandem duplication/insertion, insertion and inversion.

The requirement for both ends of an SR-supported inversion (as with PE and mixed inversions) to join the reference is to substantiate the existence of an actual inversion as there may be other kinds of inverted structural variations or artifacts present. This assessment uses relative locations

of the split partners in the sequenced read and in the alignment, and orientation information, to call an inversion event complete. The reader is referred to src/addSplitReads.py in the source code if they are interested in further details.

1.5 Variant filtering

For our case of primary alignments, variant filtering particularly addresses integrated variants that have one cluster in common and the other cluster unique to each variant (see S9 Fig). This is indicative of an atypical situation with regard to variant identification and therefore neither variant would be called unless one of them exceeds the support threshold. If both exceed the support threshold, they are addressed in the next stage. Such situations can be often seen in real sequenced samples, but are revealed upon visual analysis as artifacts. Variants that are not supported by expected clusters, for example, an inversion that is only supported by a "FF" cluster are not trustworthy as calls in any particular variant category and included simply as BND events. Also, a cluster that may have been too small to make the final support threshold filter by itself but fits cogently into an existing variant with sufficient support would now be counted and useful.

To calculate the disjointness threshold, RSVSim was used to alter the "hg19" human reference genome by introducing 500 deletions, tandem duplications, inversions and insertions (i.e., translocations and copy-paste insertions) and we used wgsim to synthetically read from this sample at coverages from 5x to 45x in steps of 10, and with a standard deviation ranging from 10 to 70 in steps of 20. SVXplorer was run on these different data sets with different minimum thresholds for support (in three different variant categories: PE, SR and "mixed" or PE+SR) and the F1 score for the identified variants was calculated against the true variant set. The threshold yielding the highest F1 score was recorded for each data set as a function of (coverage, insert length std) and used to generate a best-fit line, and all intermediate coverage values are fit by linear interpolation. The dependence on standard deviation in insert length ends up being inconsequential in this simple model.

1.6 Incorporation of depth of coverage

Coverage for each chromosome or contig is calculated using mappable regions only, which is provided as input. All final SV calls are also made using coverage calculated in the variant region using only mappable bases as far as possible. Coverage calculation seeks to use other bases only if a predefined sufficient number of mappable bases is not seen. Only uniquely aligning reads are used in calculation of coverage and reads that could be putative PCR duplicates, or refer to secondary alignments, are filtered away.

Variant-region coverage information is recorded in the INFO filed in the VCF file if a certain variant was rejected by unexpected read depth. This filter is used to enhance calls made in all SV categories except inversions.

Variants that are supported exclusively by SRs and called as possible deletions, tandem duplications or copy-paste insertions are rejected if contradicted by preset thresholds. Deletion and duplication calls that are well supported by PE alignments are not required to satisfy the preset thresholds but are not written if the VCR exceeds or falls below a slightly more liberal rejection threshold. This is because, as alluded to in an earlier section, an unmatched "FR" PE cluster that is above the coverage-determined support threshold and composed of fragments with large insert lengths is more likely a deletion than an "FF" or "RR" SR cluster.

Additionally, some routines are used to analyze the coverage of integrated variants (such as cut-paste and copy-paste insertions) in regions between relevant pairs of breakpoints and decouple them into BND events if necessary, if the SPLIT_INS parameter is turned on. As an example one "FR" and and one "RF" PE cluster may have combined to form a copy-paste insertion, but if it is now seen that the coverage between the "FR" breakpoints indicates a deletion, then the insertion call is broken into two simple BND events to be safe.

2 Supplementary Results

2.1 Simulated Data

In comparisons of performance in detection of deletions, duplications, and inversions, all methods with the exception of TARDIS exhibit a low false-positive rate. SVXplorer was uniformly the leader by a small margin ranging from $.1 - .3\%$). This is shown in S10 Fig.

When comparing the performance of SVXplorer against TIDDIT in detection of 3-breakpoint events, we found that SVXplorer is more sensitive and precise as compared to TIDDIT for this dataset. S11 FigA and S11 FigB shows the results of this simulation.

2.2 SVXplorer has high precision and sensitivity on CHM1 deletions

S12 Fig shows the distribution of deletions, duplications and inversions detected by SVXplorer for the CHM1 sample.

2.3 SVXplorer achieves high self-consistency among multiple libraries

S13 Fig shows the performance curves for SRR505885 as coverage of the library is varied. SVXplorer exhibits the highest F1 scores for all coverages considered in this study. S14 Fig shows the distribution of the size of deletions detected by SVXplorer for the ERR1341794 library. It also shows the F1 score of all methods included in this study for the various SV sizes. SVXplorer is consistently among the best callers for all sizes considered.

As seen in S15 Fig, all the callers seem to perform reasonably well on self-consistency. SVXplorer is generally best or the second-best in each category and obtains the highest normalized (by number of each SV type) self-consistency (by 5% over the second-best) with 6867 calls (LUMPY has 7012, DELLY has 6708 and MANTA has a total of 2827 SV calls). The average number of inversions called for the two libraries was 50 for SVXplorer, 30 for LUMPY, 350 for MANTA and 599 for DELLY. SVXplorer and LUMPY are much more in line with expectations compared to DELLY and MANTA.

In the AJ-Trio analysis, similar to the idea in NA12878 with sequencing libraries, we are interested in knowing whether a variant found in the child presents *any* evidence of being seen in either parent

2.3.1 NA12878 integrated variant self-consistency

The purpose of this simple check was to see if the integrated variants source breakpoints (which are usually defined by multiple clusters) are seen to at all overlap either with an “FR” or “RF” cluster, or another integrated variant source location, in the other library. This method to assess integrated variant overlap was chosen because very often only partial signatures of such variants occur (e.g., only one “FR” or one “RF” cluster for a cut-paste or copy-paste insertion) due to reasons related to coverage, homology, repeats and alignment. Therefore, if most source locations of one library overlap with another similar location in the other library, it is a good indicator of self-consistency for integrated variants. As shown in Fig 5, SVXplorer has 100% (8 of 8) and 89% (8 of 9) self-consistency for cut-paste insertions for the two libraries whereas for copy-paste insertions it has 99.5% (191 of 192) and 91% (133 of 146). The difference in self-consistency for the two libraries arises due to the fact that the sequencing, insert length distribution etc. for the two are very different. The “SRR505885” library has many more lower mapping-quality calls, particularly of “RF” alignments.

2.4 SVXplorer exhibits high self-consistency in a trio setting

We see that the percentage of calls that were in the child and not found in either parent was lowest for SVXplorer for each variant type: 3%, 10% and 7% for deletions, duplications and inversions,

respectively, whereas the second best numbers (not necessarily by the same caller) are 5%, 15% and 11% respectively. All callers perform as expected when evaluating the difference between calls shared by the child and one of the parents and those shared by both parents, with the difference never falling below 10% of the child's calls for any variant type. The number of inversions called by SVXplorer (average = 44, on same order as LUMPY's 30) is supported by other reports [?] whereas MANTA and DELLY call 288 and 556 respectively. The lower number of inversions is effected by SVXplorer's requirement to see both ends of the inversion joining the reference, by a combination of PE or SR reads. For reference, SVXplorer makes a total of 2802 calls for the child, LUMPY makes 2798, DELLY makes 2385 and MANTA makes 1235 calls. AJ trio self-consistency for the various callers is shown in S16 Fig.

2.4.1 Benchmarking of 3-breakpoint variants

In a second evaluation (Eval 2), the parents' call-sets consisted of all 3-breakpoint variant calls tagged as cut-paste insertions with known breakpoint order or as copy-paste insertions, including those listed as BND events, which passed final filters. Thus, the second evaluation additionally included BND events whose "SVSubtype" is listed as a copy-paste insertion in the BEDPE and in the PROBTYPE field under "INFO" in the VCF. An example case is a variant that was initially listed as a copy-paste insertion but whose local coverage information did not support that particular call, leading to its inclusion in the final list of variants as a BND event. So, the second set of parent calls is a superset of those used in the first evaluation leading to a more liberal assessment. A slop of 300 was used for the evaluation.

Table 1. SVXplorer's performance of 3-breakpoint variant calls on AJ Trio with BND insertions included

	Total calls	Child Calls	Overlap	"Sens"	Prec
Father	527	185	98	18.6	53.0
Mother	561	185	94	16.8	50.8
Combined	1089	185	134	17.1	72.4

The same definition of metrics is used as in Table 5 in the manuscript. Overlap now requires the child's SV type to match either the SV type or the "SVSubtype" of the parent call given the additional BND events in the latter. The sensitivity column is in quotes because we do not expect all BND events to be reproduced in related individuals or libraries (their source is uncertain). They are however used here for a more liberal assessment compared to Table 5.

As one can see, SVXplorer achieves greater than 50% precision in comparisons against one parent. Further, Table 5 records $\approx 64\%$ precision and Table 1 records $\approx 72\%$ in the combined call-set comparison. This is notable given the quality of the data and the requirement for a 3-breakpoint match. The trio comes from the same ethnic subgroup and the overlap of calls between the mother and father is $\approx 35\%$ (the father's set contained 183 calls and the mother's set had 184 calls). Of the child's 185 calls, 174 were copy-paste insertions, 9 were cut-paste insertions with confirmed breakpoint order and 2 were inverted copy-paste insertions. As a specific example, in both Table 5 and Eval 2, 4 cut-paste insertions and 3 inverted copy-paste insertions seen in the child overlapped precisely with such calls in the combined call set. The relatively small proportion of cut-paste insertions detected is not surprising given the coverage range of 27X-28X for all 3 individuals and the uncertainty in insert length, which makes it less likely for all 3 cut-paste insertion clusters to be formed and subsequently disambiguated for breakpoint order (see "Formation of paired-end clusters" in Methods for details).

3 Supplementary Algorithms

3.1 Disjointness-Based Set Cover Algorithm

This algorithm, though not directly employed, motivated the disjointness-based filter in Methods and is presented as a novel approach to estimate the best set cover. When set information is recorded, keep track of the number of elements of each set that are not shared by any other set, i.e. that are unique. Each element resides in a hash table that records the total number of sets sharing that element within the whole collection of sets. The hash also records which sets the element is a part of. The sets are also recorded elsewhere in memory with their member elements listed.

The disjointness score for a set is defined as being equal to the sum of the number of times its each element appears in any other set. For example, given sets $A = 1, 2, 3, B = 2, 3, 5$ and $C = 3, 5, 7, 8$, A has a disjointness score of 3, B has a score of 4 and C has a score of 3. The algorithm follows:

1. First, add to the set cover all the sets that have unique elements, in any order, each time removing these elements from all the respective sets they belong to. Remove all empty sets from the collection. For each element of the removed sets, remove the element's hash table entry.
2. The remaining sets now only contain elements that are non-unique. Calculate the disjointness score for the remaining sets.
3. Now, add to the set cover the set with the highest disjointness score, remove its elements from the remaining sets and the hash table of elements. Remove all empty sets from collection.
4. Repeat, steps 2 and 3 until no further sets remain.

Somewhat paradoxically, first step picks all sets that *have* unique elements in order to actually cover the universe maximally and the second step serves to pick the least number of sets to cover the remaining elements.

References

[1] Sudmant PH, Rausch T, Gardner EJ, Handsaker RE, Abyzov A, Huddleston J, et al. An integrated map of structural variation in 2,504 human genomes. Nature. 2015;526:75–81. doi:10.1038/nature15394.
