# Supplementary figures and images for: SVXplorer: Three-tier approach to identification of structural variants via sequential recombination of discordant cluster signatures
Source: PLoS Comput Biol. 2020 Mar 17;16(3):e1007737. doi: 10.1371/journal.pcbi.1007737 (PMC7100977; doi:10.1371/journal.pcbi.1007737)

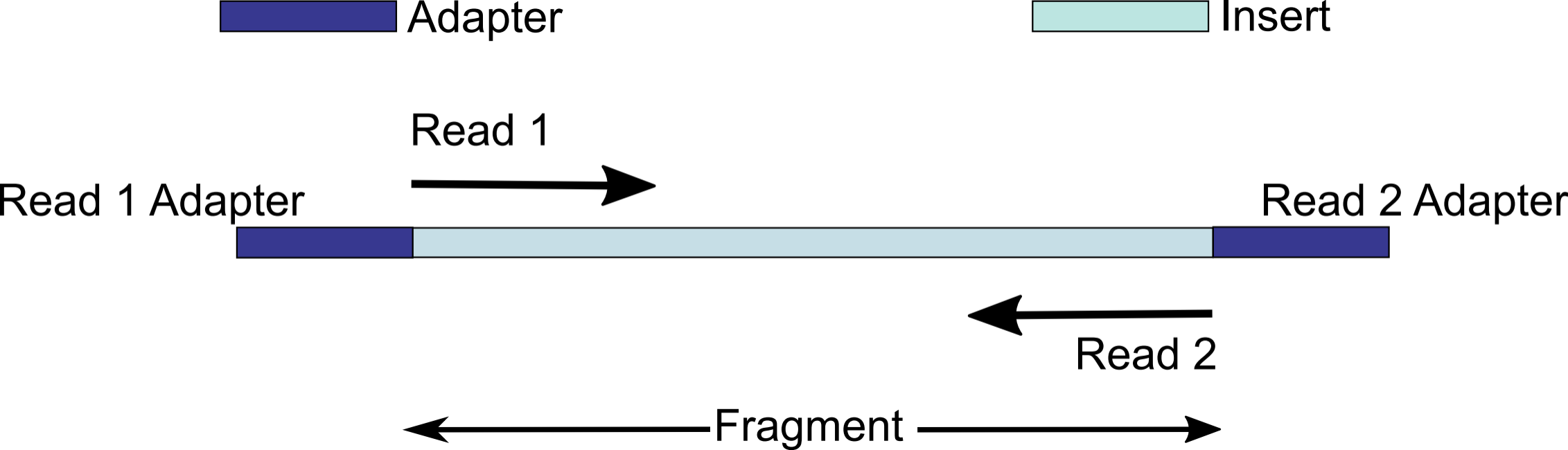

Supplement: S1 Fig — (PDF) [file pcbi.1007737.s002.pdf]

Connected Component Size Distribution for NA12878 (ERR194147)

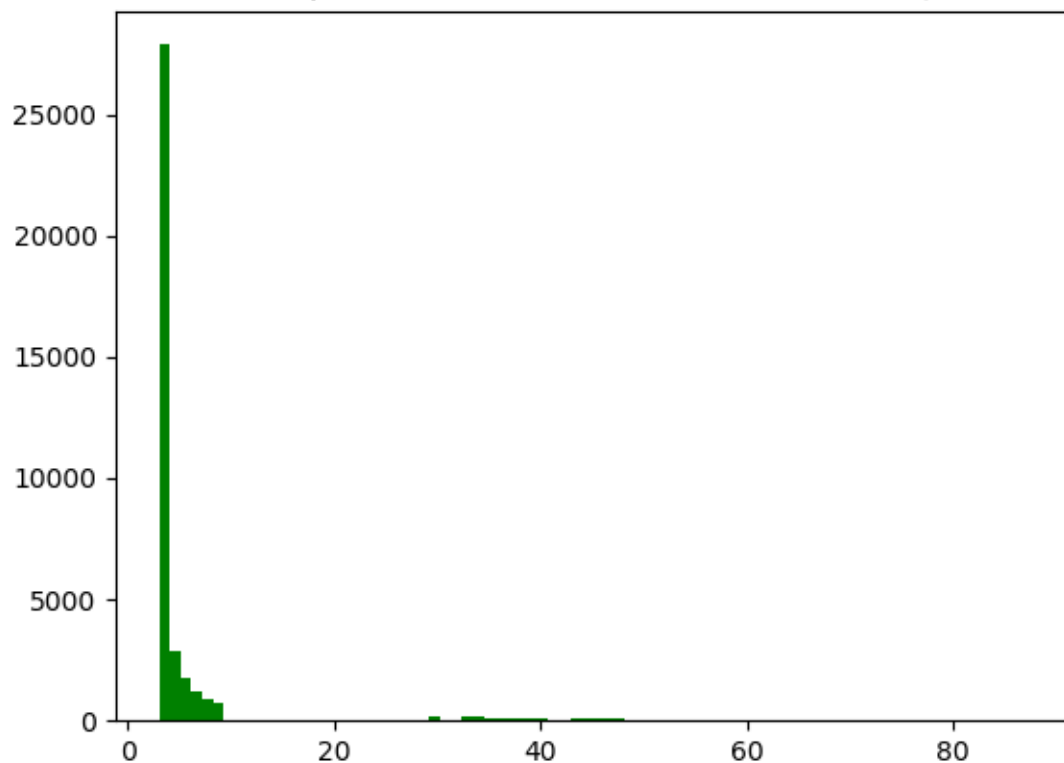

Supplement: S2 Fig — (PDF) [file pcbi.1007737.s003.pdf]

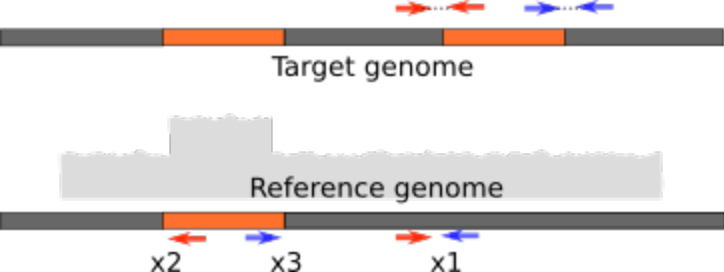

Supplement: S3 Fig — The segment in orange is duplicated downstream in the sample. The figure shows 2 distinct clusters in red and blue matching up in the reference to form a copy-paste insertion. Breakpoint 1 (x1) is defined to be the overlap of adjacent oppositely-oriented alignments from the 2 clusters, and breakpoints 2 and 3 (x2 and x3) are defined by their respective mate alignments, with x2 < x3 by convention, whether upstream or downstream from x1. (PDF) [file pcbi.1007737.s004.pdf]

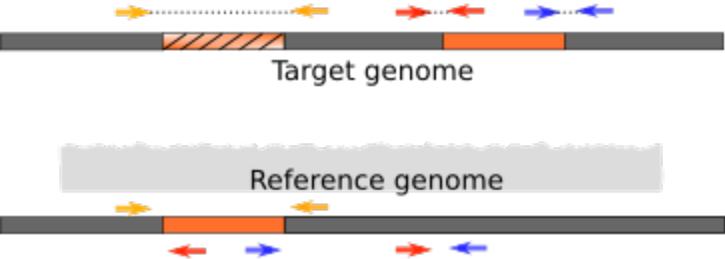

Supplement: S4 Fig — The segment in orange is deleted and pasted downstream in the sample. The figure shows 3 distinct clusters, shown in red, blue and light orange. The cluster shown in light orange is the extra “FR” cluster resulting from the deletion of the translocated segment. (PDF) [file pcbi.1007737.s005.pdf]

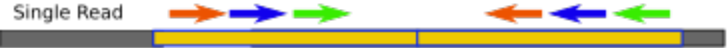

Target genome

Reference genome

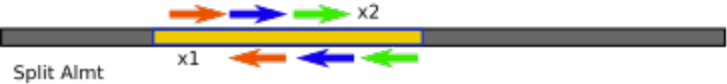

Supplement: S5 Fig — The segment in yellow is adjacently duplicated downstream in the sample. The figure thus shows sequenced fragments from a tandem duplication that align as “FR.” In such a case, the left breakpoint is defined by reverse alignments and the right breakpoint is defined by forward alignments. (PDF) [file pcbi.1007737.s006.pdf]

Single Read

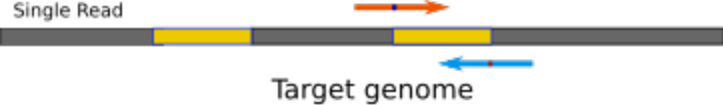

Reference genome

Split Almt

x2

x3

x1

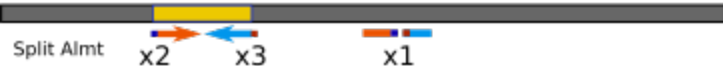

Supplement: S6 Fig — The segment in yellow is duplicated downstream in the sample. The orange read by itself would lead to a TD_I call and the blue by itself to a DEL_INS call. But together they define a copy-paste insertion consisting of 3 distinct breakpoints. (PDF) [file pcbi.1007737.s007.pdf]

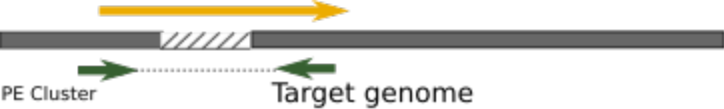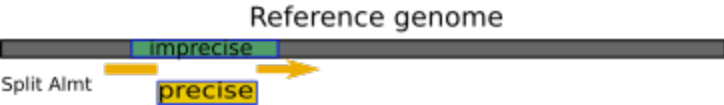

Supplement: S7 Fig — The read shown in yellow (size exaggerated in target) is split into 2 alignments in the reference close to the PE breakpoints. The segment in green is the putative PE deletion call and the segment in yellow shows revised precise breakpoints. (PDF) [file pcbi.1007737.s008.pdf]

Single Read

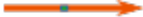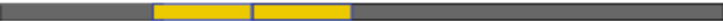

Target genome

Reference genome

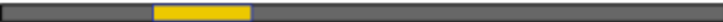

Split Almt

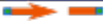

Supplement: S8 Fig — The segment in orange is tandem-duplicated downstream in the sample. The read shown in orange splits in alignment at the point shown in blue. The split partners are swapped in alignment, i.e., the head portion of the original forward-oriented read aligns in the reference to the left of the tail portion of that read. Such cases give rise to a TD_I cluster. (PDF) [file pcbi.1007737.s009.pdf]

Reference genome

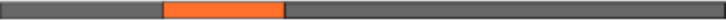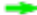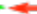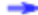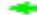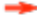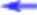

Supplement: S9 Fig — A case where 2 PE clusters each separately match up with a third cluster. The clusters in red and green match up with each other and so do the ones in red and blue, each matching pair indicating a copy-paste insertion. It is quite unlikely that both are true. This is addressed in the filtering stage. (PDF) [file pcbi.1007737.s010.pdf]

Deletion

Duplication

Inversion

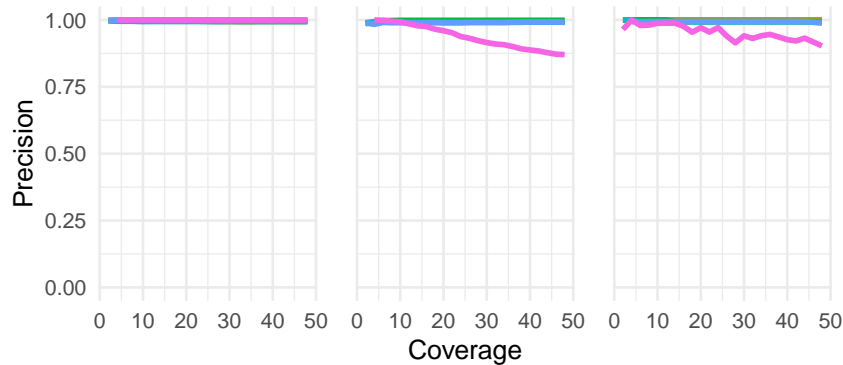

Approach

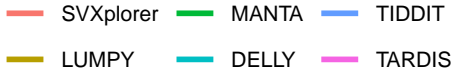

Supplement: S10 Fig — (PDF) [file pcbi.1007737.s011.pdf]

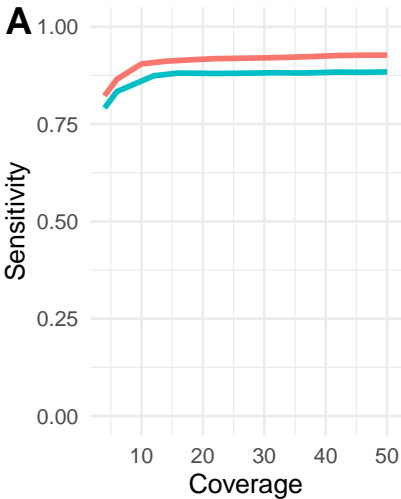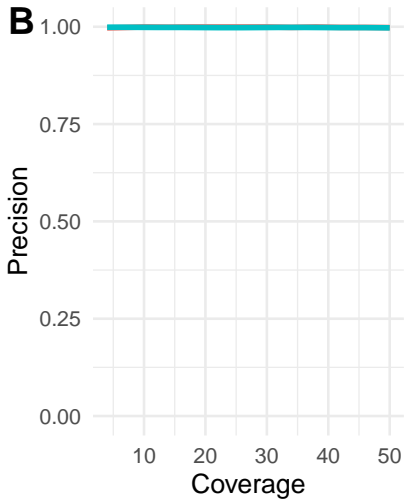

Approach — SVXplorer — TIDDIT

Supplement: S11 Fig — (A) Sensitivity with varying coverage (B) Precision with varying coverage. (PDF) [file pcbi.1007737.s012.pdf]

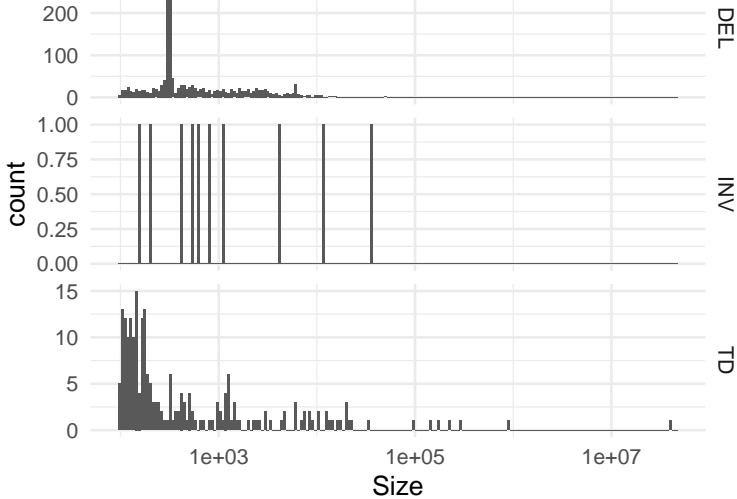

Supplement: S12 Fig — (PDF) [file pcbi.1007737.s013.pdf]

F1 score

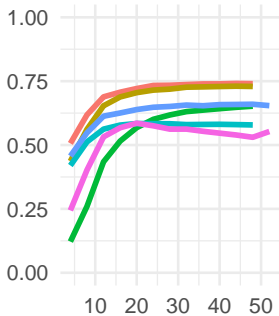

Precision

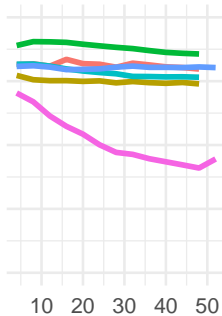

Sensitivity

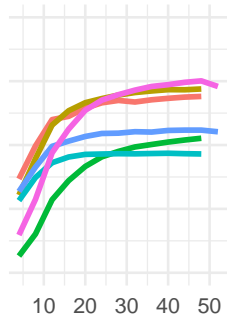

Method

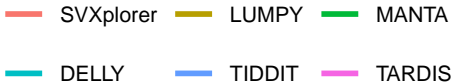

Supplement: S13 Fig — (PDF) [file pcbi.1007737.s014.pdf]

Count

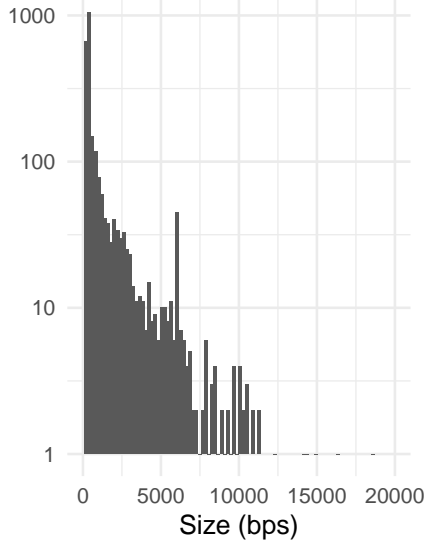

Size of deletion (bps)

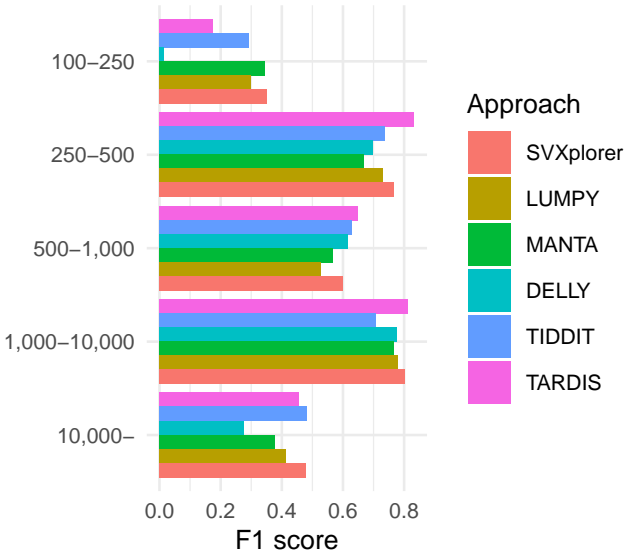

Supplement: S14 Fig — (PDF) [file pcbi.1007737.s015.pdf]

Deletion

Duplication

Inversion

SRR505885

ERR194147

approach

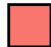

SVXplorer

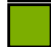

LUMPY

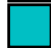

MANTA

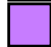

DELLY

0.00 0.25 0.50 0.75 1.000.00 0.25 0.50 0.75 1.000.00 0.25 0.50 0.75 1.00

consistency

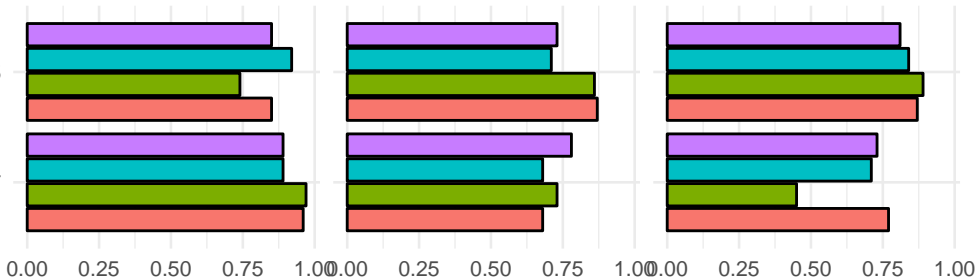

Supplement: S15 Fig — “Consistency” refers to the fraction of calls in the listed base library that were found in the other library. (PDF) [file pcbi.1007737.s016.pdf]

## DELETION

## DUPLICATION

## INVERSION

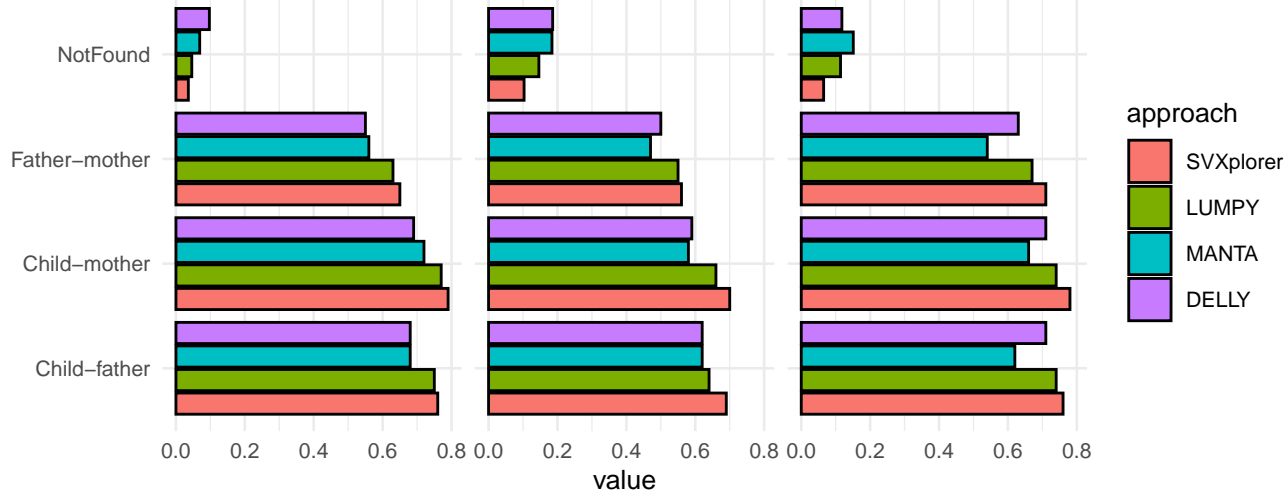

Supplement: S16 Fig — “A-B” refers to fraction of total calls made for A that were found in B. Here A or B is a placeholder for either child, father or mother. “Difference” refers to the difference between fraction of calls in common between the parents and that between child and a parent (normalized). We expect this to be large. The “not found” column shows the fraction of total calls that were made in the child that were not found in either parent. (PDF) [file pcbi.1007737.s017.pdf]
